# Therapeutic Effects of Stem Cells From Different Source on Renal Ischemia- Reperfusion Injury: A Systematic Review and Network Meta-analysis of Animal Studies

**DOI:** 10.3389/fphar.2021.713059

**Published:** 2021-09-02

**Authors:** Zhizhong Shang, Yanbiao Jiang, Xin Guan, Anan Wang, Bin Ma

**Affiliations:** ^1^ Evidence Based Medicine Center, School of Basic Medical Sciences, Lanzhou University, Lanzhou, China; ^2^ Key Laboratory of Evidence Based Medicine and Knowledge Translation of Gansu Province, Lanzhou, China; ^3^ The Second Clinical Medical School, Lanzhou University, Lanzhou, China

**Keywords:** stem cells, kidney, ischemia-reperfusion injury, systematic review, animal

## Abstract

**Objective:** Although stem cell therapy for renal ischemia-reperfusion injury (RIRI) has made immense progress in animal studies, conflicting results have been reported by the investigators. Therefore, we aimed to systematically evaluate the effects of different stem cells on renal function of animals with ischemia-reperfusion injury and to compare the efficacies of stem cells from various sources.

**Methods:** PubMed, Web of Science, Embase, Cochrane, CNKI, VIP, CBM, and WanFang Data were searched for records until April 2021. Two researchers independently conducted literature screening, data extraction, and literature quality evaluation.

**Results and conclusion:** Seventy-two animal studies were included for data analysis. Different stem cells significantly reduced serum creatinine and blood urea nitrogen levels in the early and middle stages (1 and 7 days) compared to the negative control group, however there was no significant difference in the late stage among all groups (14 days); In the early stage (1 day), the renal histopathological score in the stem cell group was significantly lower than that in the negative control group, and there was no significant difference among these stem cells. In addition, there was no significant difference between stem cell and negative control in proliferation of resident cells, however, significantly less apoptosis of resident cells than negative control. In conclusion, the results showed that stem cells from diverse sources could improve the renal function of RIRI animals. ADMSCs and MDMSCs were the most-researched stem cells, and they possibly hold the highest therapeutic potential. However, the quality of evidence included in this study is low, and there are many risks of bias. The exact efficacy of the stem cells and the requirement for further clinical studies remain unclear.

Acute kidney injury (AKI) is a common clinical syndrome, accounting for 10–15% of all hospitalized patients, and the mortality rate is as high as 20–25% (Susantitaphong et al., 2013). Additionally, in the intensive care unit, the incidence of AKI is 50–70% with greater than 50% mortality (Ronco et al., 2019; Srisawat et al., 2020). AKI is more common in older patients, patients with diabetes or vascular disease, and kidney transplant recipients (Tögel and Westenfelder, 2012; Krzywonos-Zawadzka et al., 2019). Renal ischemia-reperfusion injury (RIRI) is the main cause of AKI, resulting from oxidative stress, calcium accumulates in thecytosol, mitochondrial uncoupling, releasing of iron ions and inflammatory immune responses, all above-mentioned factors contribute to necrosis or apoptosis of tubular epithelial cells, which causes AKI (Krzywonos-Zawadzka et al., 2019). Wang et al. (Wang et al., 2019) showed that the mortality rate due to RIRI is as high as 50–80% and is increasing annually. At present, caspase inhibitors, P-selectin antagonists, antioxidant NAC, erythropoietin, ICAM-1 monoclonal antibody and ischemic preconditioning are used to alleviate RIRI, however, the specific mechanism of action and the exact efficacy of these drugs/treatments are still uncertain (Pantazi et al., 2016; Banaei and Rezagholizadeh, 2019; Kellum et al., 2021).

Stem cells originate from bone marrow, umbilical cord, and adipose tissue, etc. and are able to self-renew and have a multi-spectrum of differentiation. Stem cells play a therapeutic role through immunomodulation, anti-inflammation and tissue repair (Reinders and Rabelink, 2010; Barzegar et al., 2019; Lee et al., 2019). Stem cell transplantation can significantly improve renal tubular degeneration and necrosis, tubular formation and inflammatory cell infiltration in animals with RIRI, and reduce the levels of serum creatinine and blood urea nitrogen (Tseng et al., 2021). Stem cells have demonstrated proper efficacy for many diseases in a number of clinical trials, however, there are a limited number of clinical studies examining the role of stem cells in renal diseases (Rota et al., 2019; Chen et al., 2021). 887 clinical studies using human bone marrow mesenchymal stem cells were reported in 2019, only 5% of the studies involved kidney disease, including AKI, diabetic nephropathy, kidney transplantation and nephritis (Rota et al., 2019). Moreover, the sample size consisted only of two and four cases (Perico et al., 2011; Mudrabettu et al., 2015), a smaller sample size is difficult to test the true therapeutic effect of stem cells. In addition, the optimal source, dosage, route and frequency of administration of stem cells, and the mechanism of action are not yet clear, and the possible side effects have made it difficult to carry out large-scale clinical trials (Sávio-Silva et al., 2020).

Currently, great progress has been made in animal studies of stem cell therapy for RIRI. Animal studies have shown that stem cells can inhibit tubular degeneration and necrosis, tubular formation and inflammatory cell infiltration through homing, cell differentiation, endocrine and paracrine effects, while reducing serum creatinine and blood urea nitrogen level (Asanuma et al., 2010). However, there are still obstacles, such as low survival rate, limited targeting ability, and low transplantation efficiency (Burst et al., 2010; Qi et al., 2014; Hu et al., 2019). Additionally, stem cell therapy remains controversial. Two studies (Burst et al., 2010; Lam et al., 2017) has shown that bone marrow hematopoietic stem cells have very weak plasticity in mice with RIRI, and stem cells cannot differentiate into target cells in certain animal models. Other studies (Jiang et al., 2002; De Broe, 2005; Duffield and Bonventre, 2005; Duffield et al., 2005) have shown that hematopoietic stem cells or bone marrow mesenchymal stem cells not only do not differentiate into renal tubular epithelial cells or improve the repair process of renal tubular epithelial cells after RIRI, but in fact, activate granulocytes to aggravate renal injury. Studies (Huls et al., 2008; Burst et al., 2010) suggest that only 10% of renal tubular epithelial cells were derived from transplanted bone marrow stem cells during the repair of RIRI, and serum creatinine levels in stem cell-treated animals were similar to those that had not received stem cell therapy.

In summary, although there are many preclinical studies on stem cell therapy for RIRI, the exact efficacy of stem cell therapy remains to be explored. In addition, studies on stem cell therapy for RIRI have been carried out, but there is a lack of direct comparison between stem cells from different sources. When the outcome which lacked direct comparison, network meta-analysis has great advantages as a new method of direct and indirect comparison between different interventions and can estimate the ranking probability of interventions (Caldwell et al., 2005; Salanti et al., 2008; Chaimani et al., 2013). Therefore, it is necessary to comprehensively analyze the current relevant preclinical evidence in order to provide guidance for future animal experiments and clinical studies.

## Materials and Methods

### Data Inclusion and Exclusion Criteria

#### Diseases and Species of Animals

All animal models of RIRI were incorporated without limits on animal species.

### Interventions and Comparisons

Stem cells versus negative controls: Blank, Phosphate buffered saline solution, Excipient, Normal saline, Culture solution, Vit E, Vehicles. The stem cell therapies were not limited in type, origin, or location transplantation.

### Outcome Indicators

The main outcome indicators were serum creatinine levels, blood urea nitrogen levels, and renal histopathological changes. The secondary outcome indicators were proliferation and apoptosis of resident cells.

### Study Design

Controlled experiments were included without limiting the implementation of covert grouping or blind methods.

### Search Strategy

We searched PubMed, Ovid-Embase, The Cochrane Library, Web of Science, China National Knowledge Infrastructure (CNKI), Chinese Scientific Journal Database (CSJD-VIP), Wanfang Database, and China Biomedical Literature Database (CBM). The retrieval time represented animal studies prior to April 2021. The search terms were as follows: (stem cell OR dry cell OR derived stem cell) AND (renal OR kidney OR nephridium) AND (ischemia-reperfusion injury OR ischemia reperfusion injury OR ischemia-reperfusion OR reperfusion injury OR ischemia reperfusion). *See* Annex 1 for Chinese and English search strategies.

### Paper Selection and Data Extraction

Two trained researchers (Z. Shang and Y. Jiang) selected the papers and extracted the data in strict accordance with the inclusion/exclusion criteria, and selections were cross-checked. In the case of disagreement, a third party would make the final decision. Data was extracted according to the pre-established full-text data extraction checklist, including: *1*) Basic characteristics of included studies: authors, publication years, research types, baseline characteristics of experimental animals, sample size and modeling methods, stem cell types, sources, doses, routes of administration. *2*) Primary outcome indicators: serum creatinine level, blood urea nitrogen level, and renal histopathological changes; Secondary outcome indicators: proliferation of resident cells and apoptosis of resident cells.

### Risk Assessment of Bias

Using SYRCLE’s risk of bias tool for animal studies (Hooijmans et al., 2014), two trained researchers (X. Guan and A. Wang) independently evaluated and cross-checked the inherent risk of bias in the included studies, i.e., selection bias, performance bias, attrition bias, follow-up bias, reporting bias, and other biases from a list of 10 questions or tools. Differences in opinion were negotiated or settled by a third party (B. Ma). Answers to the assessment questions (tools) were either “yes” to indicate a low risk of bias or “no” to indicate a high risk of bias. An answer of ‘‘unclear” was assigned to items for which a “yes” or “no” answer was not clear.

### Quality Assessment of Evidence

Whether the results of the systematic review of animal studies can lead to clinical translation depends on the quality of the evidence. The Confidence in the Evidence from Reviews of Qualitative research (CERQual) tool (Guyatt et al., 2011; Lewin et al., 2015) developed by Cochrane Collaboration for the grading and evaluation of evidence assesses the quality of the following four aspects: *1*) methodological limitations, *2*) correlation, *3*) consistency of results, and *4*) adequacy of data. To assess the quality of evidence for this systematic review, we evaluated the above four criteria individually, and then the result of each criterion was combined to calculate a level of evidence of high, moderate, low, or very low (Lewin et al., 2015).

### Statistical Analysis

GeMTC-0.14.3 software based on Bayesian model was used for statistical analysis. Based on the Bayesian framework, the software uses Markov chain-Monte Carlo (Markov chain-Monte Carlo, MCMC) method to a priori and evaluate the data to achieve reticular Meta-analysis. The first iteration is set to 50,000. The deviation information criterion value (DIC) of random effect model and fixed effect model was compared to judge the fitting degree of the model. The odds ratio (OR) and the mean difference (MD) were selected as statistics for the two-category effect, and 95% confidence interval (CI) was used for both. The concordance model was used in the analysis of reticular Meta, and the difference was statistically significant. The inconsistency test uses the node analysis model, if *p* > 0.05, it means that there is no evidence to prove that there is inconsistency between direct comparison and indirect comparison. The convergence of mesh Meta is tested by potential scale reduced factor (PSRF). If PSRF is close to 1, it means that the convergence of this study is good, and the conclusion of Meta-analysis is reliable. Also, the use of Stata16.0 software for traditional Meta-analysis, and the use of network group commands for data pre-processing, to draw a comparison between the outcome indicators of the network relationship between the intervention measures.

In order to make full use of the obtained data, the main outcome indicators were merged and analyzed by nodes (1, 7, 14 days), and the secondary outcome indicators were merged and analyzed based on the data obtained in the first 5 days.

## Results

### Systematic Search Outcomes

**FIGURE 1:**
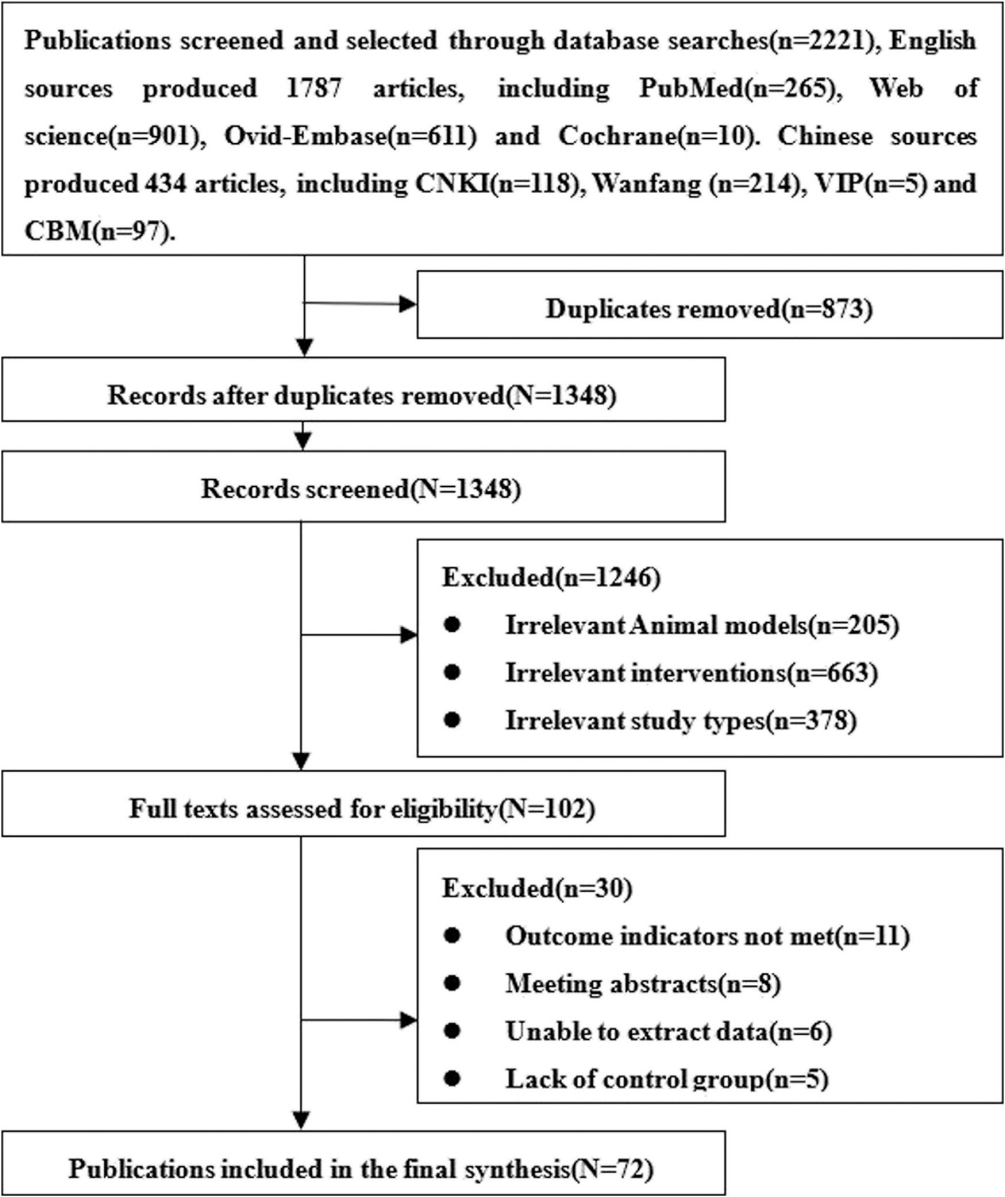
Study screening and selection process.

A total of 2,221 relevant articles were obtained, of which 434 were written in Chinese and 1787 in English. After excluding the repetitive and non-compliant studies, 72 animal studies were included ^6,22,23,35–103^, 63 English ^6,22,23,35–94^ and nine Chinese (Wang et al., 2005; Wang and Fu, 2008; Yang et al., 2008; Fang, 2009; Liu et al., 2011; Huang et al., 2012; Xie et al., 2015; Hu et al., 2016; Shi et al., 2018). The screening and selection process is outlined in Figure 1.

### Basic Information Included in the Study

The basic information from animal studies included in the current analysis is detailed in Annex 2. A total of 72 randomized controlled animal studies were included. Animal models included SD rats, Wistar rats, rabbits,129sv/C57 mice, Lewis rats, ewe and sheep. Body weights varied between (18 g) (Hattori et al., 2015) and (25 kg) (Hu et al., 2016). Ages ranged from (6 wk) (Lam et al., 2017) to (16 wk) (Hussein et al., 2016). Sample sizes ranged from (6) (Zhou et al., 2016) to (72) (Hattori et al., 2015). The modeling methods of animals were all reperfusion injury caused by clamping renal vessels, and the clamping time was between (25 min) (Wise et al., 2014) and (85 min) (Yang et al., 2008). The intervention dose of stem cells was between (1 × 10^4^) (Hu et al., 2016) and (1.5 × 10^10^) (Wang and Fu, 2008). The administration routes included artery (25%), vein (50%), intrarenal injection (18%) and intraperitoneal injection (4%).

There were great differences in the types and sources of stem cells. Adipose Tissue-Derived Mesenchymal Stem Cells (ADMSCs) were derived from SD rats (Chen et al., 2011; Gao et al., 2012; Masoud et al., 2012; Shih et al., 2013; Sheashaa et al., 2016; Zhou et al., 2016; Lam et al., 2017; Zhang et al., 2017; Changizi-Ashtiyani et al., 2020), Wistar rats (Wang et al., 2013; Hussein et al., 2016; Zhao et al., 2016; Shi et al., 2018; Awadalla et al., 2021), humans (Zhou et al., 2017; Huang et al., 2020), Fisher 344 rats (Feng et al., 2010) and C57BL/6 mice (Donizetti-Oliveira et al., 2011; Donizetti-Oliveira et al., 2012; Furuichi et al., 2012). Bone Marrow Derived Mesenchymal Stem Cells (MDMSCs) were derived from Lewis rats (Erpicum et al., 2017), Wister rats (Semedo et al., 2009; Havakhah et al., 2018), SD rats (Lange et al., 2005; Wang et al., 2005; Wang and Fu, 2008; Tögel et al., 2009; Zhuo et al., 2011; Altun et al., 2012; Huang et al., 2012; Zhuo et al., 2013; Cai et al., 2014a; Cai et al., 2014b; Zhao et al., 2014; Wang et al., 2019), C57BL/6 mice (Liu et al., 2011; Qi et al., 2014; Xie et al., 2015; Hu et al., 2016), BALB/C mice (Xing et al., 2014), Rabbits (Yang et al., 2008; Fang, 2009), Sheep (Behr et al., 2007; Behr et al., 2009) and human (Cao et al., 2010; Wise et al., 2014; Collett et al., 2017b; Zilberman-Itskovich et al., 2019). Endothelial Progenitor Cells (EPCs) were derived from SD rats (Chen et al., 2013; Collett et al., 2017a) and human cord (Liang et al., 2015). Umbilical Cord-mesenchymal Stem Cells (UC-MSCs) were derived from human (Du et al., 2012; Ryoun et al., 2014; Fahmy et al., 2017; Rodrigues et al., 2017) and Rat umbilical cords (Guo and Wang, 2018). Mesenchymal Stem Cells from Fetal Membranes (FMhMSCs) were derived from human (La Manna et al., 2011) and Lewis (MHC haplotype: RT-1l) rats (Tsuda et al., 2014). Germline Cell–derived Pluripotent Stem Cells (GPSCs) were derived from kidneys of 129sv mice (De Chiara et al., 2014). Renal Progenitor Cells (RPCs) were derived from renal tissue of SD rats (Li et al., 2015). Induced Pluripotent Stem Cells (iPSCs) were derived from the embryos of C57BL/6 mice (Lee et al., 2012). Urine-derived Stem Cells (USCs) were derived from adult urine (Tian et al., 2017; Li et al., 2020). Mesenchymal stem cells derived from human amniotic fluid (hAFSCs) (Monteiro Carvalho Mori da Cunha et al., 2015). Neural Precursor Cells (NPCs) were derived from the embryos of Wistar rats (Wang et al., 2009). Human Amnion Epithelial Cell (HAEC) was derived from human amnion (Ren et al., 2020). Stem Cells from Human Exfoliated Deciduous Teeth (SHED) were derived from human deciduous teeth (Hattori et al., 2015).

### Risk of Bias and Quality of Evidence

**FIGURE 2:**
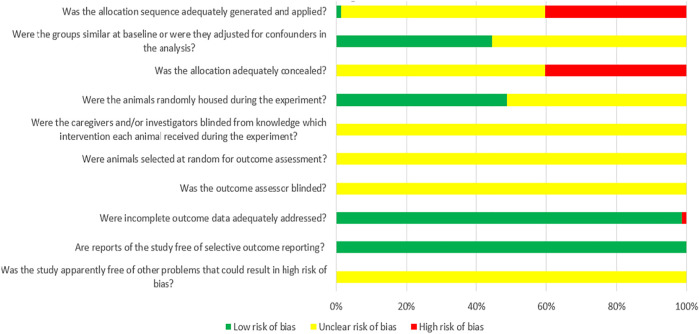
Risk of bias of each item of SYRCLE tool for overall included studies (Each risk of bias item presented as percentages across all included studies, which indicated the proportion of different level risk of bias for each item (Hooijmans et al., 2014)).

Among the 72 controlled studies included in the analysis, only one study reported specific randomized grouping methods for experimental animals (Hu et al., 2016); 32 studies reported similar baseline characteristics of the experimental animals used; none of the studies reported the use of covert grouping (Wang and Fu, 2008; Yang et al., 2008; Cao et al., 2010; Chen et al., 2011; Zhuo et al., 2011; Huang et al., 2012; Lee et al., 2012; Sadek et al., 2013; Shih et al., 2013; Zhuo et al., 2013; Cai et al., 2014b; Tsuda et al., 2014; Xing et al., 2014; Zhao et al., 2014; Hattori et al., 2015; Li et al., 2015; Monteiro Carvalho Mori da Cunha et al., 2015; Hussein et al., 2016; Zhou et al., 2016; Fahmy et al., 2017; Lam et al., 2017; Rodrigues et al., 2017; Tian et al., 2017; Zhang et al., 2017; Havakhah et al., 2018; Ko et al., 2018; Shi et al., 2018; Zilberman-Itskovich et al., 2019; Huang et al., 2020; Li et al., 2020; Ren et al., 2020; Awadalla et al., 2021); and 35 studies reported randomization of animals during experiments. (Lange et al., 2005; Semedo et al., 2009; Tögel et al., 2009; Wang et al., 2009; Zhuo et al., 2011; Donizetti-Oliveira et al., 2012; Du et al., 2012; Gao et al., 2012; Lee et al., 2012; Sadek et al., 2013; Shih et al., 2013; Wang et al., 2013; Zhuo et al., 2013; Cai et al., 2014a; Ryoun et al., 2014; Gupta et al., 2015; Hattori et al., 2015; Li et al., 2015; Liang et al., 2015; Monteiro Carvalho Mori da Cunha et al., 2015; Zhao et al., 2016; Zhou et al., 2016; Collett et al., 2017a; Fahmy et al., 2017; Rodrigues et al., 2017; Tian et al., 2017; Zhang et al., 2017; Zhou et al., 2017; Guo and Wang, 2018; Havakhah et al., 2018; Ko et al., 2018; Zilberman-Itskovich et al., 2019; Changizi-Ashtiyani et al., 2020; Li et al., 2020); None of the studies reported blinding of animal breeders and researchers. None of the studies reported the methods for selecting animals in the evaluation of results. All studies did not apply blind method to outcome evaluators. Only one experimental animal in study died after modeling (Lam et al., 2017). Although we were unable to obtain the research proposals for any of the studies, all of the expected results were reported. The results of the bias risk assessment of the 72 studies included in our analysis are detailed in Figure 2.

The results from assessing the quality of evidence showed “low” or “very low” quality in the five outcome indicators. The reasons for the poor quality of evidence included the lack of internal authenticity of the original research, the inconsistency of the research results, and the inability to quantitatively combine the data.

### Meta-analysis Results

#### Serum Creatinine Levels

**FIGURE 3:**
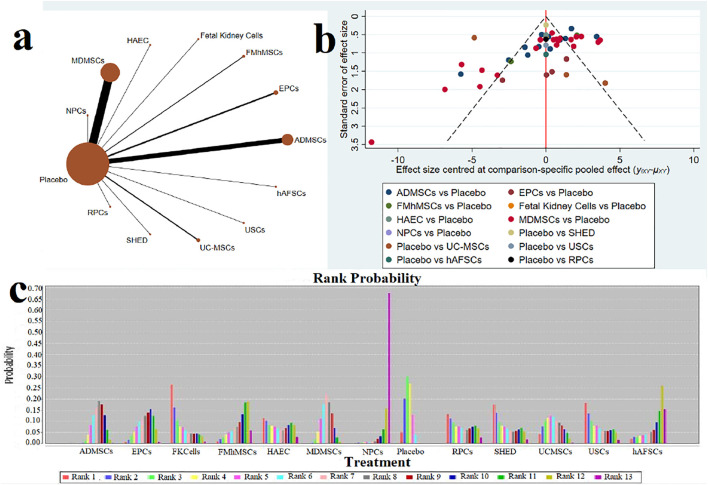
Network Meta-analysis of serum creatinine levels at 1 day after administration **(A)** Evidence network diagram; **(B)** The comparison-correction funnel plot; **(C)** The ranking results).

**FIGURE 4:**
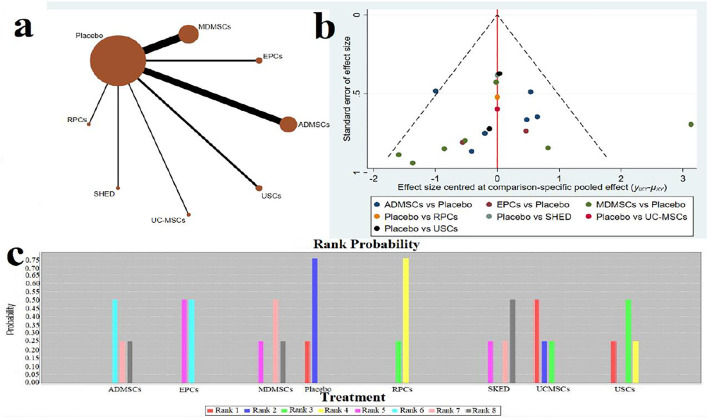
Network Meta-analysis of serum creatinine levels at 7 days after administration **(A)** Evidence network diagram; **(B)** The comparison-correction funnel plot; **(C)** The ranking results).

**FIGURE 5:**
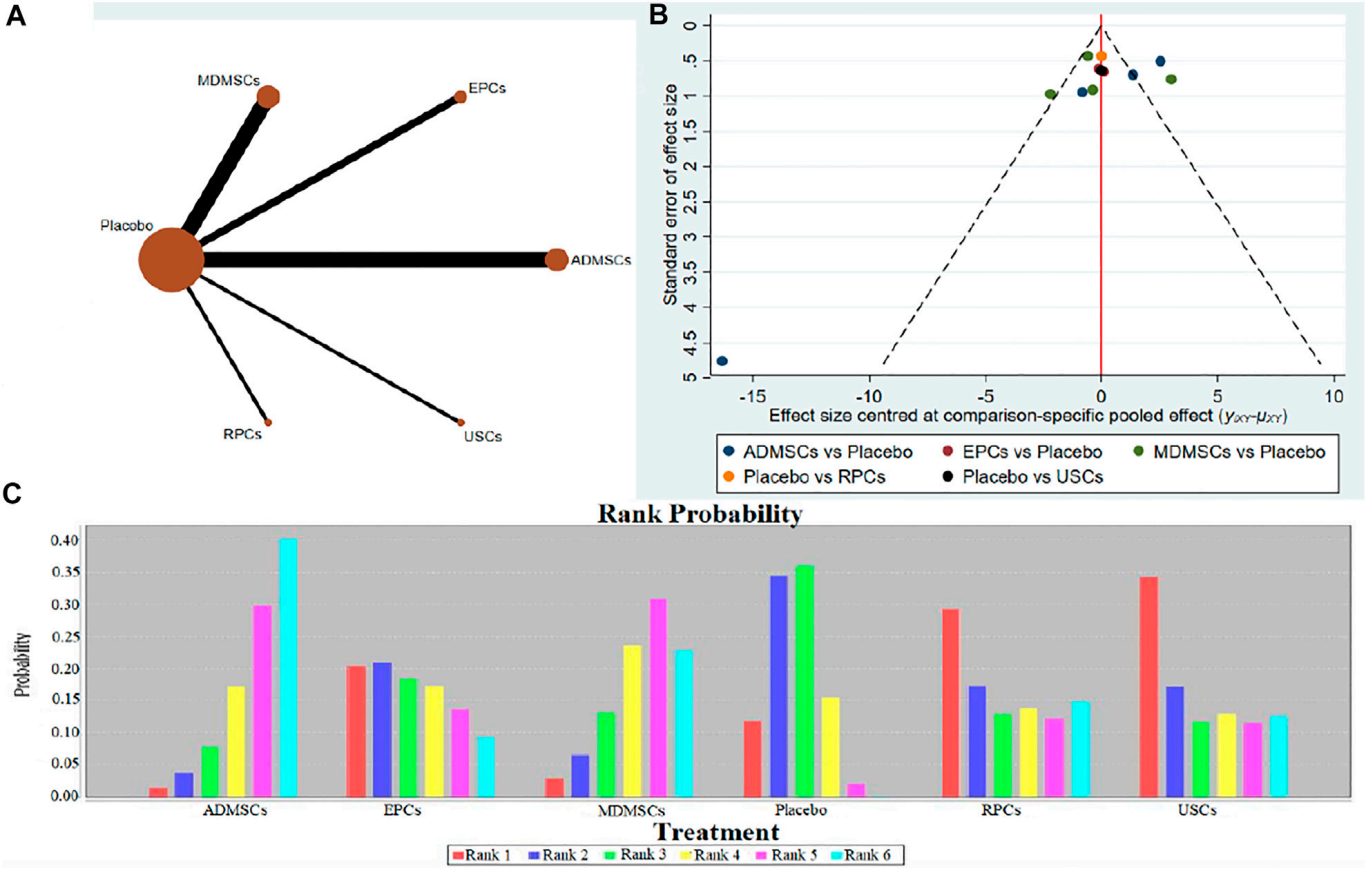
Network Meta-analysis of serum creatinine levels at 14 days after administration **(A)** Evidence network diagram; **(B)** The comparison-correction funnel plot; **(C)** The ranking results).

①1 day after administration: 49 studies were included for data analysis^23,35,36,39–42,44,45,47,49–51,53–60,62,66,68,72–75,77–80,82–84,86–99^. The results of traditional meta-analysis were detailed in Table 1, showed that serum creatinine levels in various of stem cell groups were lower than those in the negative control group. The results of network meta-analysis showed that MDMSCs and ADMSCs had the highest number of studies (Figure 3A); There was no significant difference in the level of serum creatinine among different of stem cells, as detailed in Table 2; The comparison-correction funnel plot was asymmetric, suggesting that publication bias and small sample effect might exist (Figure 3B). The ranking results showed that Fetal Kidney Cells might be one of the most effective to reduce serum creatinine level (Figure 3C); ②7 days after administration: 20 studies were included for data analysis (Donizetti-Oliveira et al., 2012; Changizi-Ashtiyani et al., 2020; Ryoun et al., 2014; Furuichi et al., 2012; Liang et al., 2015; Gupta et al., 2015; Rodrigues et al., 2017; Tian et al., 2017; Havakhah et al., 2018; La Manna et al., 2011; Hussein et al., 2016; Awadalla et al., 2021; Zhao et al., 2014; De Chiara et al., 2014; Fahmy et al., 2017; Zhao et al., 2016; Li et al., 2015; Liu et al., 2011; Fang, 2009; Yang et al., 2008). The results of traditional meta-analysis were detailed in Table 3, showed that that serum creatinine levels in various of stem cell groups were lower than those in the negative control group. The results of network meta-analysis showed that MDMSCs and ADMSCs had the highest number of studies (Figure 4A); ADMSCs showed better therapeutic effect than other types of stem cells, as detailed in Table 4; The comparison-correction funnel plot was asymmetric, suggesting that publication bias and small sample effect might exist (Figure 4B); The ranking results showed that UCMSCs might be one of the most effective stem cells to reduce serum creatinine level (Figure 4C). ③14 days after administration: 12 studies were included for data analysis (Yang et al., 2008; Fang, 2009; Liu et al., 2011; Donizetti-Oliveira et al., 2012; Gao et al., 2012; De Chiara et al., 2014; Ryoun et al., 2014; Lewin et al., 2015; Li et al., 2015; Rodrigues et al., 2017; Havakhah et al., 2018; Awadalla et al., 2021). The results of traditional meta-analysis were detailed in Table 5, showed that no significant difference in serum creatinine levels between stem cell groups and negative control group except RPCs. The network meta-analysis showed that MDMSCs and ADMSCs had the highest number of studies (Figure 5A); There was no significant difference in the level of serum creatinine among different stem cell groups, as detailed in Table 6; The comparison-correction funnel plot was asymmetric, suggesting that publication bias and small sample effect might exist (Figure 5B). The ranking results showed that USCs may be one of the most effective stem cells to reduce serum creatinine level in all of stem cells (Figure 5C).

**TABLE 1:** Traditional Meta-analysis results of serum creatinine levels between stem cell groups and the control group at 1 day after administration.

Intervention measures	Research quality	SMD [95% CI]	*I*^2^/%	Z	*p*
ADMSCs VS Negative control	12	−2.164 [−3.159 to −1.169]	85.4%	4.26	0.000
MDMSCs VS Negative control	21	−1.718 [−2.597 to −0.838]	85.8%	3.38	0.000
EPCs VS Negative control	4	−5.914 [−7.648 to −4.180]	30.1%	6.69	0.232
UC-MSCs VS Negative control	3	−5.275 [−11.147 to 0.597]	93.7%	1.76	0.000
hAFSCs VS Negative control	2	−3.309 [−5.350 to −1.268]	—	3.18	—
FMhMSCs VS Negative control	1	−3.314 [−7.684 to 0.879]	91.0%	1.49	0.001
Fetal Kidney Cells VS Negative control	1	0.166 [−0.968 to 1.300]	—	0.29	—
USCs VS Negative control	1	−1.877 [−3.421 to −0.334]	—	2.38	—
NPCs VS Negative control	1	−2.561 [−3.773 to −1.350]	—	4.14	—
HAEC VS Negative control	1	−2.014 [−3.013 to −1.015]	—	3.95	—
SHED VS Negative control	1	−0.424 [−0.892 to 0.043]	—	1.78	—
RPCs VS Negative control	1	−4.174 [−5.398 to −2.950]	—	6.68	—

**TABLE 2:** Network Meta-analysis results of serum creatinine levels in stem cell groups at 1 day after administration.

ADMSCs	−0.06 (−0.97 to 0.87)	0.65 (−1.02 to 2.34)	−0.32 (−1.63 to 0.96)	0.21 (−1.44 to 1.88)	0.08 (−0.52 to 0.70)	−1.52 (−3.33 to 0.33)	0.31 (−1.37 to 1.94)	0.44 (−1.26 to 2.11)	0.26 (−0.76 to 1.31)	0.45 (−1.24 to 2.14)	−0.53 (−2.24 to 1.15)
0.06 (−0.87 to 0.97)	EPCs	0.71 (−1.11 to 2.52)	−0.26 (−1.72 to 1.16)	0.27 (−1.50 to 2.11)	0.15 (−0.74 to 1.02)	−1.47 (−3.41 to 0.50)	0.35 (−1.42 to 2.11)	0.51 (−1.34 to 2.31)	0.33 (−0.95 to to 1.53)	0.50 (−1.34 to 2.31)	-0.47 (−2.32 to 1.34)
−0.65 (−2.34 to 1.02)	−0.71 (−2.52 to 1.11)	FKCells	−0.98 (−3.02 to 1.04)	−0.45 (−2.71 to 1.86)	−0.56 (−2.24 to 1.07)	−2.17 (−4.54 to 0.22)	−0.34 (−2.65 to 1.90)	−0.21 (−2.56 to 2.04)	−0.38 (−2.26 to 1.46)	−0.22 (−2.50 to 2.09)	−1.18 (−3.50 to 1.11)
0.32 (−0.96 to 1.63)	0.26 (−1.16 to 1.72)	0.98 (−1.04 to 3.02)	FMhMSCs	0.53 (−1.47 to 2.55)	0.41 (−0.85 to 1.68)	−1.19 (−3.33 to 0.93)	0.63 (−1.44 to 2.66)	0.75 (−1.25 to 2.83)	0.59 (−0.92 to 2.13)	0.76 (−1.27 to 2.75)	−0.20 (−2.25 to 1.83)
−0.21 (−1.88 to 1.44)	−0.27 (−2.11 to 1.50)	0.45 (−1.86 to 2.71)	−0.53 (−2.55 to 1.47)	HAEC	−0.13 (−1.79 to 1.51)	−1.75 (−4.09 to 0.65)	0.10 (−2.26 to 2.32)	0.22 (−2.10 to 2.49)	0.05 (−1.78 to 1.91)	0.24 (−2.08 to 2.51)	−0.74 (−3.06 to 1.56)
−0.08 (−0.70 to 0.52)	−0.15 (−1.02 to 0.74)	0.56 (−1.07 to 2.24)	−0.41 (−1.68 to 0.85)	0.13 (−1.51 to 1.79)	MDMSCs	−1.60 (−3.39 to 0.22)	0.22 (−1.47 to 1.86)	0.35 (−1.35 to 2.04)	0.18 (−0.80 to 1.18)	0.37 (−1.30 to 2.02)	−0.62 (−2.29 to 1.03)
1.52 (−0.33 to 3.33)	1.47 (−0.50 to 3.41)	2.17 (−0.22 to 4.54)	1.19 (−0.93 to 3.33)	1.75 (−0.65 to 4.09)	1.60 (−0.22 to 3.39)	NPCs	1.82 (−0.58 to 4.17)	1.94 (−0.46 to 4.36)	1.78 (−0.25 to 3.78)	1.95 (−0.45 to 4.37)	0.99 (−1.46 to 3.33)
−0.31 (−1.94 to 1.37)	−0.35 (−2.11 to 1.42)	0.34 (−1.90 to 2.65)	−0.63 (−2.66 to 1.44)	−0.10 (−2.32 to 2.26)	−0.22 (−1.86 to 1.47)	−1.82 (−4.17 to 0.58)	RPCs	0.13 (−2.09 to 2.42)	−0.04 (−1.90 to 1.83)	0.14 (−2.14 to 2.43)	−0.84 (−3.14 to 1.46)
−0.44 (−2.11 to 1.26)	−0.51 (−2.31 to 1.34)	0.21 (−2.04 to 2.56)	−0.75 (−2.83 to 1.25)	−0.22 (−2.49 to 2.10)	−0.35 (−2.04 to 1.35)	−1.94 (−4.36 to 0.46)	−0.13 (−2.42 to 2.09)	SHED	−0.17 (−2.06 to 1.71)	0.02 (−2.29 to 2.29)	−0.97 (−3.26 to 1.36)
−0.26 (−1.31 to 0.76)	−0.33 (−1.53 to 0.95)	0.38 (−1.46 to 2.26)	−0.59 (−2.13 to 0.92)	−0.05 (−1.91 to 1.78)	−0.18 (−1.18 to 0.80)	−1.78 (−3.78 to 0.25)	0.04 (−1.83 to 1.90)	0.17 (−1.71 to 2.06)	UCMSCs	0.19 (−1.68 to 2.04)	−0.80 (−2.74 to 1.07)
−0.45 (−2.14 to 1.24)	−0.50 (−2.31 to 1.34)	0.22 (−2.09 to 2.50)	−0.76 (−2.75 to 1.27)	−0.24 (−2.51 to 2.08)	−0.37 (−2.02 to 1.30)	−1.95 (−4.37 to 0.45)	−0.14 (−2.43 to 2.14)	−0.02 (−2.29 to 2.29)	−0.19 (−2.04 to 1.68)	USCs	−0.98 (−3.27 to 1.31)
0.53 (−1.15 to 2.24)	0.47 (−1.34 to 2.32)	1.18 (−1.11 to 3.50)	0.20 (−1.83 to 2.25)	0.74 (−1.56 to 3.06)	0.62 (−1.03 to 2.29)	−0.99 (−3.33 to 1.46)	0.84 (−1.46 to 3.14)	0.97 (−1.36 to 3.26)	0.80 (−1.07 to 2.74)	0.98 (−1.31 to 3.27)	hAFSCs

**TABLE 3:** Traditional Meta-analysis results of serum creatinine levels between stem cell groups and the control group at 7 days after administration.

Intervention measures	Research quality	SMD [95% CI]	*I*^2^/%	Z	*p*
ADMSCs VS Negative control	6	−1.939 [−2.541 to −1.336]	31.7%	6.31	0.198
MDMSCs VS Negative control	7	−1.407 [−2.587 to −0.227]	78.4%	2.34	0.000
EPCs VS Negative control	2	−1.969 [−3.036 to −0.903]	0.0%	3.62	0.350
USCs VS Negative control	2	−1.515 [−2.163 to −0.867]	0.0%	4.58	0.842
UC-MSCs VS Negative control	1	−0.665 [−1.834 to 0.503]	—	1.12	—
SHED VS Negative control	1	−0.800 [−1.547 to −0.052]	—	2.10	—
RPCs VS Negative control	1	−3.130 [−4.150 to −2.111]	—	6.02	—

**TABLE 4:** Network Meta-analysis results of serum creatinine levels in stem cell groups at 7 days after administration.

ADMSCs	0.11 (0.00 to 0.47)	-0.03 (-0.18 to 0.51)	0.39 (0.16 to 0.62)	0.33 (0.10 to 0.51)	-0.04 (-0.27 to 0.39)	0.40 (0.13 to 0.63)	0.34 (0.16 to 0.58)
−0.11 (−0.47 to −0.00)	EPCs	−0.14 (−0.18 to 0.04)	0.20 (0.14 to 0.33)	0.14 (0.04 to 0.27)	−0.16 (−0.51 to 0.18)	0.22 (0.13 to 0.30)	0.17 (0.11 to 0.27)
0.03 (−0.51 to 0.18)	0.14 (−0.04 to 0.18)	MDMSCs	0.35 (0.10 to 0.48)	0.29 (0.00 to 0.42)	−0.01 (−0.55 to 0.33)	0.37 (0.12 to 0.43)	0.32 (0.07 to 0.42)
−0.39 (−0.62 to −0.16)	−0.20 (−0.33 to −0.14)	−0.35 (−0.48 to −0.10)	Placebo	−0.06 (−0.10 to −0.05)	−0.36 (−0.66 to −0.15)	−0.01 (−0.05 to 0.06)	−0.04 (−0.06 to 0.00)
−0.33 (−0.51 to −0.10)	−0.14 (−0.27 to −0.04)	−0.29 (−0.42 to −0.00)	0.06 (0.05 to 0.10)	RPCs	−0.30 (−0.55 to −0.10)	0.07 (0.00 to 0.12)	0.03 (−0.01 to 0.07)
0.04 (−0.39 to 0.27)	0.16 (−0.18 to 0.51)	0.01 (−0.33 to 0.55)	0.36 (0.15 to 0.66)	0.30 (0.10 to 0.55)	SHED	0.37 (0.10 to 0.67)	0.33 (0.09 to 0.62)
−0.40 (−0.63 to −0.13)	−0.22 (−0.30 to −0.13)	−0.37 (−0.43 to −0.12)	0.01 (−0.06 to 0.05)	−0.07 (−0.12 to −0.00)	−0.37 (−0.67 to −0.10)	UCMSCs	−0.03 (−0.11 to 0.03)
−0.34 (−0.58 to −0.16)	−0.17 (−0.27 to −0.11)	−0.32 (−0.42 to −0.07)	0.04 (−0.00 to 0.06)	−0.03 (−0.07 to 0.01)	−0.33 (−0.62 to −0.09)	0.03 (−0.03 to 0.11)	USCs

**TABLE 5:** Traditional Meta-analysis results of serum creatinine levels between stem cell groups and the control group at 14 days after administration.

Intervention measures	Research quality	SMD [95% CI]	*I*^2^/%	Z	*p*
ADMSCs VS Negative control	4	−2.537 [−5.120 to 0.046]	87.7%	1.93	0.000
MDMSCs VS Negative control	4	−0.780 [−2.750 to 1.190]	86.7%	0.78	0.000
EPCs VS Negative control	2	−0.774 [−1.648 to 0.100]	0.0%	1.74	0.820
USCs VS Negative control	0	0.221 [−1.023 to 1.465]	—	0.35	—
RPCs VS Negative control	0	−2.000 [−2.832 to −1.168]	—	4.71	—

**TABLE 6:** Network Meta-analysis results of serum creatinine levels in stem cell groups at 14 days after administration.

ADMSCs	0.41 (−0.68 to 1.52)	0.13 (−0.81 to 1.01)	0.45 (−1.00 to 1.89)	0.50 (−0.88 to 1.91)
−0.41 (−1.52 to 0.68)	EPCs	−0.30 (−1.42 to 0.80)	0.02 (−1.56 to 1.61)	0.08 (−1.46 to 1.62)
−0.13 (−1.01 to 0.81)	0.30 (−0.80 to 1.42)	MDMSCs	0.32 (−1.12 to 1.77)	0.38 (−1.01 to 1.81)
−0.45 (−1.89 to 1.00)	−0.02 (−1.61 to 1.56)	−0.32 (−1.77 to 1.12)	RPCs	0.06 (−1.71 to 1.90)
−0.50 (−1.91 to 0.88)	−0.08 (−1.62 to 1.46)	−0.38 (−1.81 to 1.01)	−0.06 (−1.90 to 1.71)	USCs

### Blood Urea Nitrogen Levels

**FIGURE 6:**
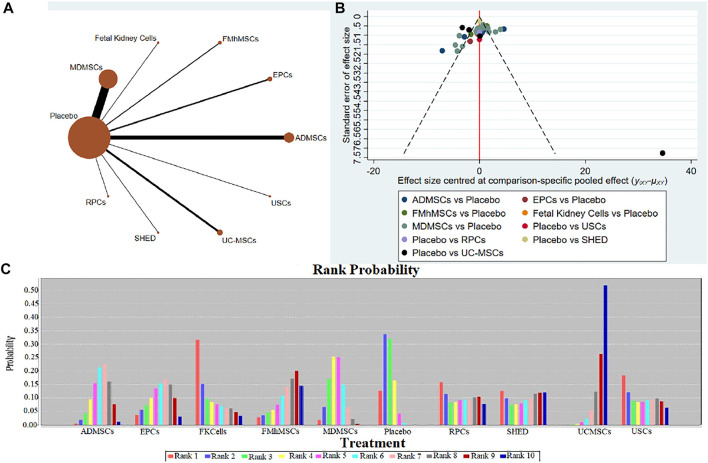
Network Meta-analysis of blood urea nitrogen level at 1 day after administration **(A)** Evidence network diagram; **(B)** The comparison-correction funnel plot; **(C)** The ranking results).

**FIGURE 7:**
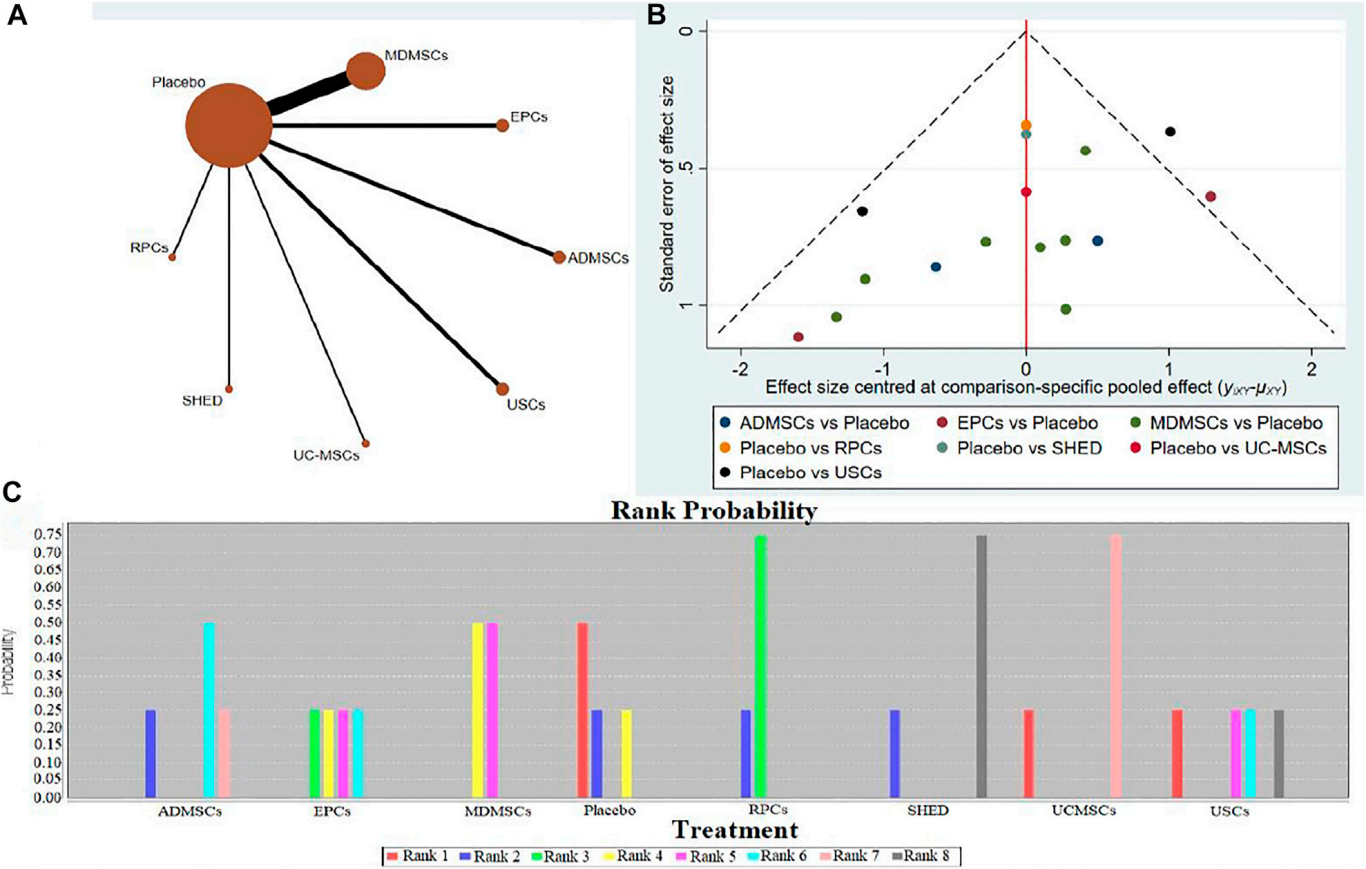
Network Meta-analysis of blood urea nitrogen level at 7 days after administration **(A)** Evidence network diagram; **(B)** The comparison-correction funnel plot; **(C)** The ranking results).

**FIGURE 8:**
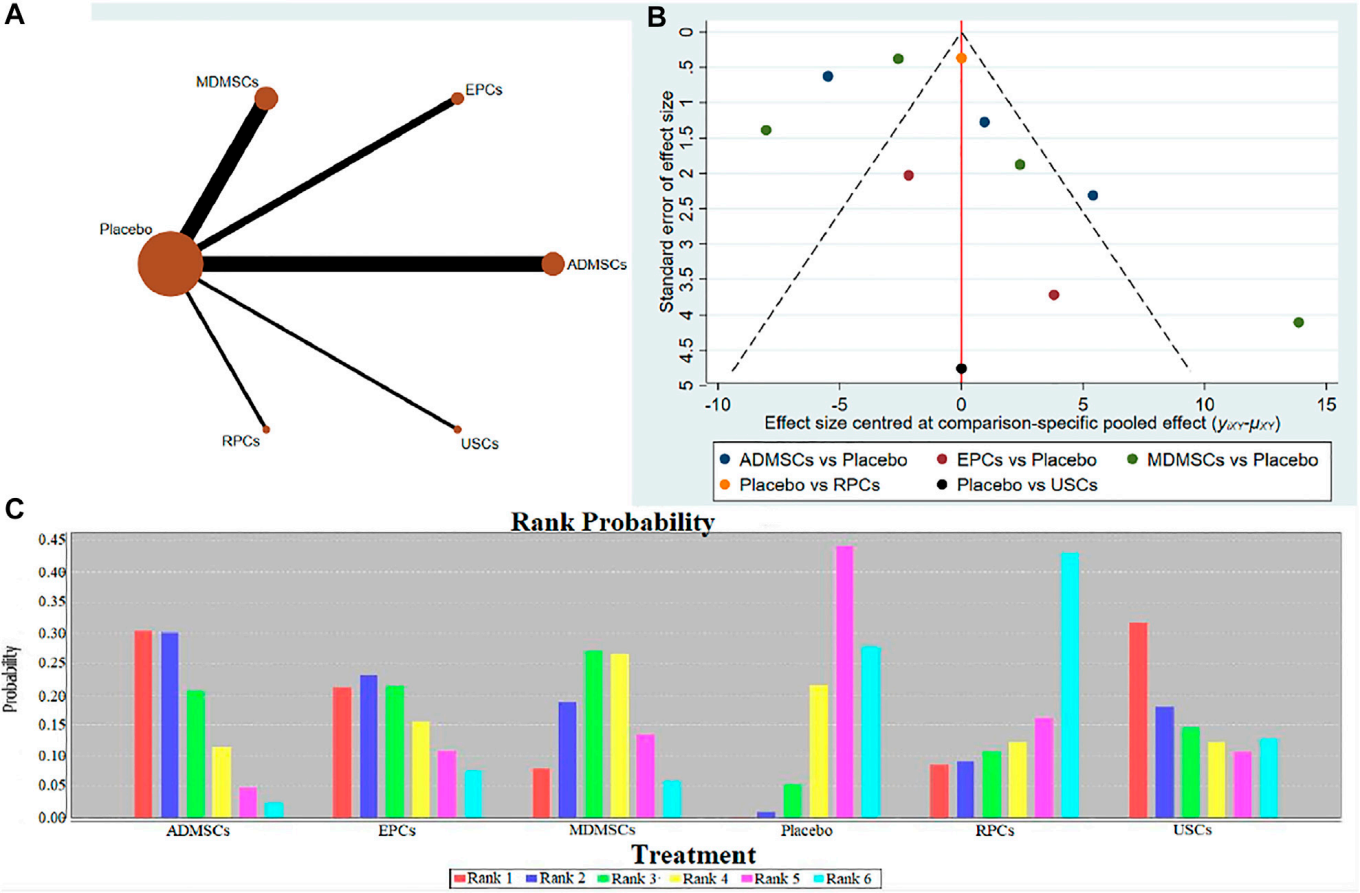
Network Meta-analysis of blood urea nitrogen level at 14 days after administration **(A)** Evidence network diagram; **(B)** The comparison-correction funnel plot; **(C)** The ranking results).

①1 day after administration: 36 studies were included for data analysis (Chen et al., 2011; Erpicum et al., 2017; Guo and Wang, 2018; Ryoun et al., 2014; Bo et al., 2013; Zhuo et al., 2013; Liang et al., 2015; Behr et al., 2009; Gupta et al., 2015; Sadek et al., 2013; Rodrigues et al., 2017; Tian et al., 2017; Havakhah et al., 2018; Behr et al., 2007; Cai et al., 2014b; Zhuo et al., 2011; Hu et al., 2013; Cao et al., 2010; La Manna et al., 2011; Xing et al., 2014; Hussein et al., 2016; Awadalla et al., 2021; Zhou et al., 2016; Zhao et al., 2014; Chen et al., 2013; Donizetti-Oliveira et al., 2011; Fahmy et al., 2017; Hattori et al., 2015; Zhao et al., 2016; Tsuda et al., 2014; Li et al., 2015; Gao et al., 2012; Altun et al., 2012; Liu et al., 2011; Wang et al., 2005; Wang and Fu, 2008). The results of traditional meta-analysis were detailed in Table 7, showed that blood urea nitrogen levels in different types of stem cell groups were lower than those in the negative control group. The results of network meta-analysis showed that MDMSCs and ADMSCs had the highest number of studies (Figure 6A); There was no significant difference in blood urea nitrogen levels among different types of stem cell groups, as detailed in Table 8; The comparison-correction funnel plot was asymmetric, suggesting that publication bias and small sample effect might exist (Figure 6B); The ranking results showed that Fetal Kidney Cells might be one of the most effective to reduce blood urea nitrogen level (Figure 6C). ②7 days after administration: 16 studies were included for data analysis (Changizi-Ashtiyani et al., 2020; Ryoun et al., 2014; Furuichi et al., 2012; Liang et al., 2015; Rodrigues et al., 2017; Tian et al., 2017; Havakhah et al., 2018; La Manna et al., 2011; Hussein et al., 2016; Awadalla et al., 2021; De Chiara et al., 2014; Fahmy et al., 2017; Li et al., 2015; Liu et al., 2011; Fang, 2009; Yang et al., 2008). The results of traditional meta-analysis were detailed in Table 9, the blood urea nitrogen levels of MDMSCs and ADMSCs were lower than those of the negative control group, and there was no significant difference in serum creatinine levels between the other groups and the negative control group. The results of network meta-analysis showed that MDMSCs and ADMSCs had the highest number of studies (Figure 7A); The blood urea nitrogen levels in EPCs and MDMSCs groups were lower than those in RPCs group, and the difference was statistically significant. The blood urea nitrogen level of SHED group was lower than that of UCMSCs, and the difference was statistically significant. There was no significant difference in blood urea nitrogen levels among the other stem cell groups, as shown in Table 10; The comparison-correction funnel plot was asymmetric, suggesting that publication bias and small sample effect might exist (Figure 7B). The ranking results showed that UCMSCs and USCs might be one of the most effective stem cells to reduce blood urea nitrogen level in all stem cells (Figure 7C). ③14 days after administration: 11 studies were included for data analysis (Yang et al., 2008; Fang, 2009; Liu et al., 2011; Gao et al., 2012; De Chiara et al., 2014; Ryoun et al., 2014; Lewin et al., 2015; Li et al., 2015; Rodrigues et al., 2017; Havakhah et al., 2018; Awadalla et al., 2021). The results of traditional meta-analysis were detailed in Table 11, showed that except RPCs, there was no significant difference in serum creatinine levels between the other types of stem cell groups and the negative control group. The results of network meta-analysis showed that MDMSCs and ADMSCs had the highest number of studies (Figure 8A); There was no significant difference in blood urea nitrogen levels between different stem cell groups, as detailed in Table 12; The comparison-correction funnel plot was asymmetric, suggesting that publication bias and small sample effect might exist (Figure 8B); The ranking results showed that USCs might be one of the most effective stem cells to reduce blood urea nitrogen level in all stem cells (Figure 8C).

**TABLE 7:** Traditional Meta-analysis results of blood urea nitrogen level between stem cell groups and the control group at 1 day after administration.

Intervention measures	Research quality	SMD [95% CI]	*I*^2^/%	Z	*p*
MDMSCs VS Negative control	16	−1.715 [−2.623 to −0.806]	83.0%	3.70	0.000
ADMSCs VS Negative control	7	−2.239 [−3.880 to −0.597	89.0%	2.67	0.000
EPCs VS Negative control	3	−2.902 [−4.594 to −1.211]	62.7%	3.36	0.068
UC-MSCs VS Negative control	4	−3.788 [−7.164 to −0.412]	91.1%	2.20	0.000
FMhMSCs VS Negative control	2	−2.372 [−5.331 to 0.587]	87.5%	1.57	0.005
Fetal Kidney Cells VS Negative control	1	−0.474 [−1.624 to 0.677]	—	0.81	—
USCs VS Negative control	1	−4.185 [−6.581 to −1.789]	—	3.42	—
SHED VS Negative control	1	−0.479 [−0.948 to −0.010]	—	2.00	—
RPCs VS Negative control	1	−6.170 [−7.824 to −4.515]	—	7.31	—

**TABLE 8:** Network Meta-analysis results of blood urea nitrogen levels in stem cell groups at 1 day after administration.

ADMSCs	1.97 (−49.44 to 53.74)	26.95 (−52.73 to 103.57)	−11.45 (−74.96 to 52.21)	14.96 (−18.06 to 49.75)	9.27 (−65.99 to 89.39)	3.10 (−77.83 to 82.82)	−36.69 (−83.84 to 11.13)	12.80 (−65.90 to 89.21)
−1.97 (−53.74 to 49.44)	EPCs	25.08 (−61.72 to 108.86)	−13.43 (−83.81 to 58.34)	13.52 (−33.70 to 59.33)	7.12 (−75.40 to 92.27)	0.43 (−87.07 to 88.68)	−38.76 (−95.41 to 18.07)	11.00 (−73.14 to 95.45)
−26.95 (−103.57 to 52.73)	−25.08 (−108.86 to 61.72)	FKCells	−37.77 (−129.95 to 54.07)	−11.94 (−86.37 to 66.06)	−17.69 (−119.99 to 88.68)	−23.96 (−129.06 to 81.40)	−63.78 (−145.37 to 20.06)	−14.33 (−114.59 to 90.79)
11.45 (−52.21 to 74.96)	13.43 (−58.34 to 83.81)	37.77 (−54.07 to 129.95)	FMhMSCs	26.16 (−33.91 to 86.59)	20.59 (−71.64 to 112.29)	14.15 (−78.92 to 108.39)	−25.60 (−93.73 to 43.99)	23.53 (−67.54 to 116.79)
−14.96 (−49.75 to 18.06)	−13.52 (−59.33 to 33.70)	11.94 (−66.06 to 86.37)	−26.16 (−86.59 to 33.91)	MDMSCs	−5.48 (−79.55 to 69.85)	−12.23 (−91.81 to 64.87)	−51.80 (−94.45 to −10.11)	−2.33 (−76.97 to 71.59)
−9.27 (−89.39 to 65.99)	−7.12 (−92.27 to 75.40)	17.69 (−88.68 to 119.99)	−20.59 (−112.29 to 71.64)	5.48 (−69.85 to 79.55)	RPCs	−6.63 (−111.32 to 97.91)	−46.16 (−128.57 to 36.84)	3.21 (−101.29 to 105.48)
−3.10 (−82.82 to 77.83)	−0.43 (−88.68 to 87.07)	23.96 (−81.40 to 129.06)	−14.15 (−108.39 to 78.92)	12.23 (−64.87 to 91.81)	6.63 (−97.91 to 111.32)	SHED	−39.76 (−124.25 to 47.47)	9.71 (−93.02 to 117.39)
36.69 (−11.13 to 83.84)	38.76 (−18.07 to 95.41)	63.78 (−20.06 to 145.37)	25.60 (−43.99 to 93.73)	51.80 (10.11 to 94.45)	46.16 (−36.84 to 128.57)	39.76 (−47.47 to 124.25)	UCMSCs	49.37 (−33.27 to 131.38)
−12.80 (−89.21 to 65.90)	−11.00 (−95.45 to 73.14)	14.33 (−90.79 to 114.59)	−23.53 (−116.79 to 67.54)	2.33 (−71.59 to 76.97)	−3.21 (−105.48 to 101.29)	−9.71 (−117.39 to 93.02)	−49.37 (−131.38 to 33.27)	USCs

**TABLE 9:** Traditional Meta-analysis results of blood urea nitrogen level between stem cell groups and the control group at 7 days after administration.

Intervention measures	Research quality	SMD [95% CI]	*I*^2^/%	Z	*p*
MDMSCs VS Negative control	7	−1.978 [−2.513 to −1.443]	0.0%	7.25	0.616
ADMSCs VS Negative control	2	−2.210 [−3.329 to −1.092]	0.0%	3.87	0.324
EPCs VS Negative control	2	−2.076 [−4.890 to 0.739]	80.7%	1.45	0.023
USCs VS Negative control	2	−0.443 [−2.553 to 1.666]	87.8%	0.41	0.004
UC-MSCs VS Negative control	1	−0.443 [−1.591 to 0.705]	—	0.76	—
SHED VS Negative control	1	−0.651 [−1.389 to 0.086]	—	1.73	—
RPCs VS Negative control	1	−0.102 [−0.775 to 0.570]	—	0.3	—

**TABLE 10:** Network Meta-analysis results of blood urea nitrogen levels in stem cell groups at 7 days after administration.

ADMSCs	8.36 (−10.55 to 21.25)	9.68 (−6.20 to 18.43)	18.68 (−1.81 to 31.20)	−79.70 (−111.18 to 24.15)	−10.48 (−23.69 to 26.89)	−2.16 (−30.34 to 34.36)
−8.36 (−21.25 to 10.55)	EPCs	0.76 (−3.09 to 5.73)	9.34 (6.73 to 13.89)	−85.06 (−127.33 to 23.56)	−18.87 (−33.77 to 26.31)	−4.96 (−30.92 to 13.11)
−9.68 (−18.43 to 6.20)	−0.76 (−5.73 to 3.09)	MDMSCs	8.99 (4.38 to 12.77)	−85.82 (−124.24 to 17.83)	−19.63 (-30.68 to 20.57)	−5.59 (−36.65 to 15.93)
−18.68 (−31.20 to 1.81)	−9.34 (−13.89 to −6.73)	−8.99 (−12.77 to −4.38)	RPCs	−94.40 (−134.06 to 9.67)	−28.21 (−40.50 to 12.42)	−12.69 (−44.81 to 3.16)
79.70 (−24.15 to 111.18)	85.06 (−23.56 to 127.33)	85.82 (−17.83 to 124.24)	94.40 (−9.67 to 134.06)	SHED	66.19 (2.74 to 93.56)	89.33 (−54.48 to 121.96)
10.48 (−26.89 to 23.69)	18.87 (−26.31 to 33.77)	19.63 (−20.57 to 30.68)	28.21 (−12.42 to 40.50)	−66.19 (−93.56 to −2.74)	UCMSCs	18.50 (−57.23 to 37.70)
2.16 (−34.36 to 30.34)	4.96 (−13.11 to 30.92)	5.59 (−15.93 to 36.65)	12.69 (−3.16 to 44.81)	−89.33 (−121.96 to 54.48)	−18.50 (−37.70 to 57.23)	USCs

**TABLE 11:** Traditional Meta-analysis results of blood urea nitrogen level between stem cell groups and the control group at 14 days after administration.

Intervention measures	Research quality	SMD [95% CI]	*I*^2^/%	Z	*p*
ADMSCs VS Negative control	3	3.483 [−2.649 to 9.616]	94.6%	1.11	0.000
MDMSCs VS Negative control	4	2.377 [−2.686 to 7.441]	92.2%	0.92	0.000
EPCs VS Negative control	2	10.857 [5.227 to 16.488]	49.7%	3.78	0.159
USCs VS Negative control	1	18.871 [9.541 to 28.200]	—	3.96	—
RPCs VS Negative control	1	−1.072 [−1.793 to −0.350]	—	2.91	—

**TABLE 12:** Network Meta-analysis results of blood urea nitrogen levels in stem cell groups at 14 days after administration.

ADMSCs	−8.25 (−84.25 to 64.42)	−16.35 (−78.56 to 46.73)	−36.30 (−134.29 to 58.22)	−5.91 (−102.64 to 90.05)
8.25 (−64.42 to 84.25)	EPCs	−7.76 (−79.10 to 64.14)	−28.83 (−127.94 to 72.30)	2.65 (−97.79 to 104.54)
16.35 (−46.73 to 78.56)	7.76 (−64.14 to 79.10)	MDMSCs	−19.88 (−112.19 to 70.78)	10.43 (−80.63 to 101.59)
36.30 (−58.22 to 134.29)	28.83 (−72.30 to 127.94)	19.88 (−70.78 to 112.19)	RPCs	30.91 (−83.99 to 146.51)
5.91 (−90.05 to 102.64)	-2.65 (−104.54 to 97.79)	−10.43 (−101.59 to 80.63)	−30.91 (−146.51 to 83.99)	USCs

#### Renal Histopathological Changes

**FIGURE 9:**
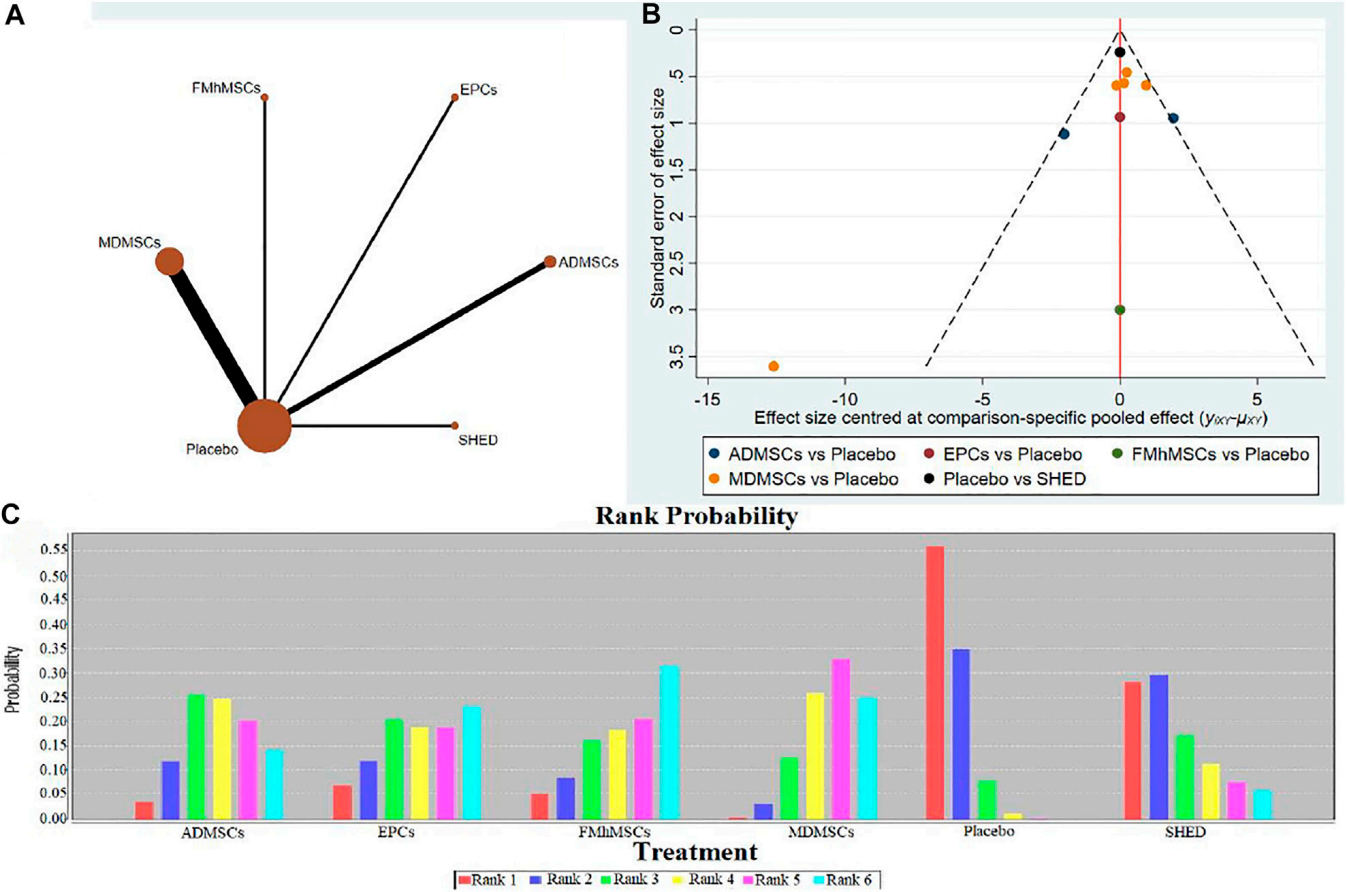
Network Meta-analysis of renal histopathological changes **(A)** Evidence network diagram; **(B)** The comparison-correction funnel plot; **(C)** The ranking results).

10 studies were included for data analysis (Zhuo et al., 2011; Chen et al., 2013; Zhuo et al., 2013; Cai et al., 2014a; Cai et al., 2014b; Tsuda et al., 2014; Hattori et al., 2015; Zhao et al., 2016; Zhou et al., 2016; Havakhah et al., 2018). The results of traditional meta-analysis were detailed in Table 13, showed that the histological scores of different stem cell groups were lower than those of the negative control group. The results of network meta-analysis showed that MDMSCs and ADMSCs had the highest number of studies (Figure 9A); There was no significant difference in histological scores between different stem cell groups, as detailed in Table 14; The comparison-correction funnel plot was basically symmetrical, suggesting that publication bias was less likely (Figure 9B); The ranking results showed that SHED might be one of the most effective to reduce renal tissue damage (Figure 9C).

**TABLE 13:** Traditional Meta-analysis results of histological scores between stem cell groups and the control group in early stage after administration.

Intervention measures	Research quality	SMD [95% CI]	*I*^2^/%	Z	*p*
MDMSCs VS Negative control	5	−1.598 [−2.728 to −0.469]	72.5%	3.52	0.006
ADMSCs VS Negative control	2	−4.116 [−8.005 to −0.228]	86.4%	2.77	0.007
EPCs VS Negative control	1	−3.289 [−5.121 to −1.456]	—	2.07	—
FMhMSCs VS Negative control	1	−10.097 [−15.975 to −4.218]	—	3.37	—
SHED VS Negative control	1	−0.615 [−1.088 to −0.142]	—	2.55	—

**TABLE 14:** Network Meta-analysis results of histological Scores in stem cell groups in early stage after administration.

ADMSCs	−0.07 (−2.42 to 2.16)	−0.24 (−2.50 to 2.04)	−0.28 (−1.88 to 1.37)	0.62 (−1.73 to 2.86)
0.07 (−2.16 to 2.42)	EPCs	−0.17 (−2.72 to 2.54)	−0.21 (−2.25 to 1.98)	0.69 (−1.93 to 3.39)
0.24 (−2.04 to 2.50)	0.17 (−2.54 to 2.72)	FMhMSCs	−0.05 (−2.04 to 2.05)	0.86 (−1.86 to 3.40)
0.28 (−1.37 to 1.88)	0.21 (−1.98 to 2.25)	0.05 (−2.05 to 2.04)	MDMSCs	0.90 (−1.21 to 2.92)
−0.62 (−2.86 to 1.73)	−0.69 (−3.39 to 1.93)	−0.86 (−3.40 to 1.86)	−0.90 (−2.92 to 1.21)	SHED

### Proliferation of Resident Cells

**FIGURE 10:**
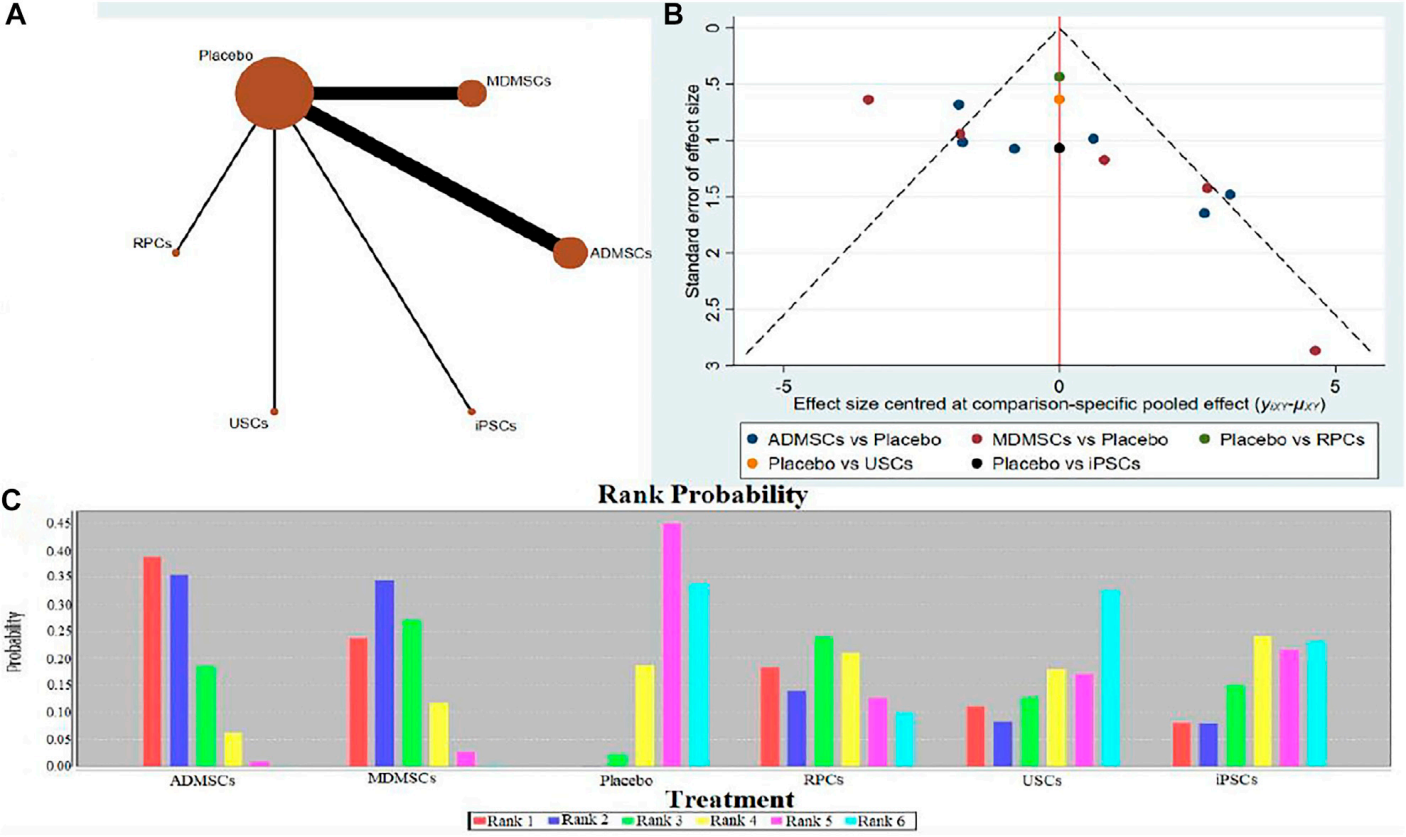
Network Meta-analysis of proliferation of resident cells in stem cell group and negative control group in early stage after administration **(A)** Evidence network diagram; (**B)** The comparison-correction funnel plot; **(C)** The ranking results).

14 studies were included for data analysis (Behr et al., 2009; Cao et al., 2010; Donizetti-Oliveira et al., 2011; Liu et al., 2011; Donizetti-Oliveira et al., 2012; Gao et al., 2012; Lee et al., 2012; Sadek et al., 2013; Shih et al., 2013; Wise et al., 2014; Li et al., 2015; Zhou et al., 2016; Tian et al., 2017; Zhou et al., 2017). The results of traditional meta-analysis are detailed in Table 15, showed that there was no significant difference in proliferation of resident cells between different types of stem cells and negative control group. The results of network meta-analysis showed that MDMSCs and ADMSCs had the highest number of studies (Figure 10A); There was no significant difference in proliferation of resident cells among different stem cells groups, as detailed in Table 16; The comparison-correction funnel plot was basically symmetrical, suggesting that publication bias was less likely (Figure 10B); The ranking results showed that proliferation of resident cells in ADMSCs group was the highest (Figure 10C);

**TABLE 15:** Traditional Meta-analysis results of proliferation of resident cells between stem cell groups and the control group in early stage after administration.

Intervention measures	Research quality	SMD [95% CI]	*I*^2^/%	Z	*p*
ADMSCs VS Negative control	6	4.282 [−2.734 to 5.829]	69.0%	5.42	0.006
MDMSCs VS Negative control	5	3.138 [0.530 to 5.746]	85.0%	2.36	0.000
USCs VS Negative control	1	0.240 [−1.005 to 1.485]	—	0.38	—
iPSCs VS Negative control	1	5.425 [3.330 to 7.519]	—	5.08	—
RPCs VS Negative control	1	2.156 [1.301 to 3.012]	—	4.94	—

**TABLE 16:** Network Meta-analysis results of proliferation of resident cells in stem cell groups in early stage after administration.

ADMSCs	−2.05 (−17.44, 12.56)	−7.17 (−33.40, 18.97)	−13.95 (−45.47, 16.84)	−12.79 (−39.17, 13.46)
2.05 (−12.56 to 17.44)	MDMSCs	−5.05 (−31.73, 22.31)	−11.90 (−43.13 to 19.37)	−10.80 (−36.94 to 16.54)
7.17 (−18.97 to 33.40)	5.05 (−22.31 to 31.73)	RPCs	−6.73 (−44.90 to 30.62)	−5.74 (−40.57 to 29.04)
13.95 (−16.84 to 45.47)	11.90 (−19.37 to 43.13)	6.73 (−30.62 to 44.90)	USCs	1.09 (−36.88 to 40.15)
12.79 (−13.46 to 39.17)	10.80 (−16.54 to 36.94)	5.74 (−29.04 to 40.57)	−1.09 (−40.15 to 36.88)	iPSCs

### Apoptosis of Resident Cells

**FIGURE 11:**
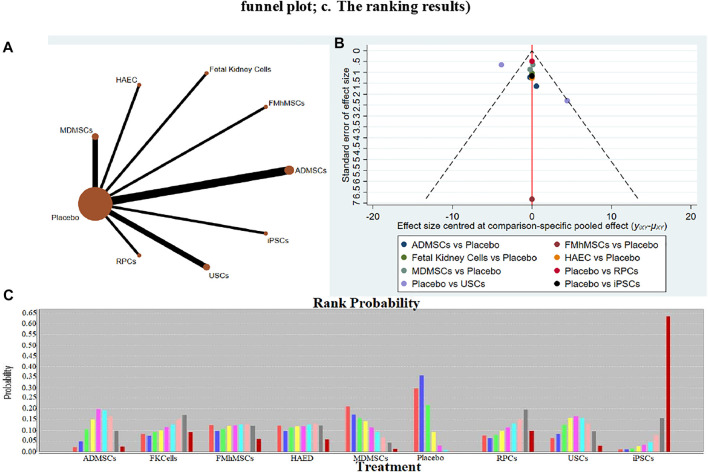
Network Meta-analysis of apoptosis of resident cells in stem cell group and negative control group in early stage after administration **(A)** Evidence network diagram; **(B)** The comparison-correction funnel plot; **(C)** The ranking results).

12 studies were included for data analysis (Behr et al., 2009; Liu et al., 2011; Lee et al., 2012; Shih et al., 2013; Tsuda et al., 2014; Gupta et al., 2015; Li et al., 2015; Zhou et al., 2016; Tian et al., 2017; Zhou et al., 2017; Li et al., 2020; Ren et al., 2020). The results of traditional meta-analysis were detailed in Table 17, showed that there was no significant difference in apoptosis of resident cells between the MDMSCs, USCs, FMhMSCs treatment groups and the negative control group. The degree of apoptosis of resident cells in the other stem cell groups was lower than that in the negative control group, and the difference was statistically significant. The results of network meta-analysis showed that MDMSCs and ADMSCs had the highest number of studies (Figure 11A); There was no significant difference in apoptosis of resident cells among different stem cells groups, as detailed in Table 18. The comparison-correction funnel plot was basically symmetrical, suggesting that publication bias was less likely (Figure 11B); The ranking results showed that the apoptosis of resident cells in MDMSCs group was the lowest (Figure 11C).

**TABLE 17:** Traditional Meta-analysis results of apoptosis of resident cells between stem cell groups and the control group in early stage after administration.

Intervention measures	Research quality	SMD [95% CI]	*I*^2^/%	Z	*p*
ADMSCs VS Negative control	3	−5.661 [−7.104 to −4.219]	0.0%	7.69	0.923
MDMSCs VS Negative control	2	−0.569 [−1.578 to 0.441]	0.0%	1.10	0.743
USCs VS Negative control	2	−4.408 [−12.502 to 3.685]	91.7%	1.07	0.001
FMhMSCs VS Negative control	1	−23.527 [−36.911 to 10.144]	—	3.45	—
Fetal Kidney Cells VS Negative control	1	−3.863 [−5.900 to −1.827]	—	3.72	—
iPSCs VS Negative control	1	−6.019 [−8.299 to −3.738]	—	5.17	—
HAEC VS Negative control	1	−7.904 [−10.374 to −5.434]	—	6.27	—

**TABLE 18:** Network Meta-analysis results of apoptosis of resident cells in stem cell groups in early stage after administration.

ADMSCs	−1.27 (−31.56, 29.97)	1.53 (−29.72, 32.37)	1.46 (−28.26, 33.07)	6.70 (−19.20, 32.72)	−2.20 (−34.13, 28.74)	1.42 (−22.79, 26.42)	−17.07 (−48.47, 14.29)
1.27 (−29.97 to 31.56)	FKCells	2.69 (−35.78 to 41.06)	2.59 (−35.16 to 41.42)	7.89 (−25.64 to 41.30)	−0.77 (−39.60 to 37.70)	2.63 (−30.77 to 35.83)	−15.76 (−55.00 to 22.84)
−1.53 (−32.37 to 29.72)	−2.69 (−41.06 to 35.78)	FMhMSCs	0.05 (−37.44 to 38.18)	5.31 (−28.56 to 38.48)	−3.68 (−41.93 to 34.34)	−0.16 (−32.91 to 33.84)	−18.53 (−56.73 to 20.19)
−1.46 (−33.07 to 28.26)	−2.59 (−41.42 to 35.16)	−0.05 (−38.18 to 37.44)	HAEC	5.33 (−28.86 to 38.17)	−3.63 (−41.75 to 31.99)	−0.03 (−32.97 to 32.91)	−18.52 (−57.78 to 19.55)
−6.70 (−32.72 to 19.20)	−7.89 (−41.30 to 25.64)	−5.31 (−38.48 to 28.56)	−5.33 (−38.17 to 28.86)	MDMSCs	−8.97 (−42.87 to 24.75)	−5.20 (−32.33 to 23.17)	−23.72 (−56.91 to 10.28)
2.20 (−28.74 to 34.13)	0.77 (−37.70 to 39.60)	3.68 (−34.34 to 41.93)	3.63 (−31.99 to 41.75)	8.97 (−24.75 to 42.87)	RPCs	3.49 (−28.52 to 37.59)	−14.67 (−53.51 to 22.91)
−1.42 (−26.42 to 22.79)	−2.63 (−35.83 to 30.77)	0.16 (−33.84 to 32.91)	0.03 (−32.91 to 32.97)	5.20 (−23.17 to 32.33)	−3.49 (−37.59 to 28.52)	USCs	−18.31 (−52.12 to 14.32)
17.07 (−14.29 to 48.47)	15.76 (−22.84 to 55.00)	18.53 (−20.19 to 56.73)	18.52 (−19.55 to 57.78)	23.72 (−10.28 to 56.91)	14.67 (−22.91 to 53.51)	18.31 (−14.32 to 52.12)	iPSCs

## Discussion

As a potential therapeutic method, stem cells from different sources hold promise in animal RIRI models. However, there are great variations in stem cell types, sources, treatment dose, treatment time, and outcome criteria. Moreover, there is a lack of direct comparison of stem cells from different sources. Therefore, it is not clear which stem cells are the most effective. Hence, we conducted a systematic review and network meta-analysis of 72 animal experiments that met the inclusion criteria, comprehensively evaluated the efficacy of stem cell therapy for RIRI, and compared the therapeutic potential of stem cells from different sources to determine the optimal stem cell therapy.

### Summary of Evidence

The results of meta-analysis by direct comparison indicated that stem cells could significantly improve renal function of RIRI animals in the early stage (1 and 7 days), compared to negative controls. On the contrary, in the late stage (14 days), there was no significant difference between the two groups of animals, which may be related to the self-renewal and repair of renal tissue cells and the gradual recovery of renal function. In addition, our study demonstrated that there was no significant difference in proliferation of resident cells between the two groups in the early stage of treatment. This observation could be related to severe necrosis of the renal tissue cells caused by ischemia-reperfusion injury and stem cells need a period of migration after injection to reach the target organ for repairing the injury. In the late stage, the migration of stem cells to the target organs and the self-renewal and repair of renal tissue cells could have led to significant differences in cell proliferation between the two groups. Studies have also shown that with the passage of time, stem cells are cleared by the immune system, resulting in their inability to play a role in promoting cell proliferation in the late stage. However, owing to the fact that most studies have not reported proliferation of resident cells, it is still unclear whether stem cells can promote cell proliferation in this stage. Therefore, future research should pay attention to the proliferation level of resident cells in order to verify the real efficacy of stem cells. Furthermore, the degree of apoptosis was significantly lower in the stem cell treatment group than that in the negative control group, suggesting that stem cells can reduce early cell death in RIRI animals.

Through network meta-analysis, 13 stem cells from different sources were indirectly compared. The results alluded that ADMSCs had the best therapeutic effect in reducing serum creatinine. Nonetheless, there was no significant difference among the stem cells from different sources in reducing blood urea nitrogen and renal histopathological score. The ranking probability diagram showed that fetal kidney cells may be the most promising ones for treatment, but their credibility may be affected by small sample sizes because only one fetal kidney cell-related study was included (Behr et al., 2009; Braun et al., 2013). The detection of cell proliferation and apoptosis revealed that there was no significant difference in the therapeutic roles of various stem cells in promoting cell proliferation and alleviating apoptosis. The sorting probability map alluded that ADMSCs (cell proliferation) and MDMSCs (apoptosis) have better therapeutic potential than the other stem cells. Studies have shown that the paracrine characteristics of ADMSCs are different from those of MDMSCs, with the former exhibiting stronger anti-inflammatory and immune regulation functions than the latter (Banas et al., 2008). In addition, ADMSCs have higher availability and lower invasiveness, which may be advantageous in future clinical applications. Studies have shown that the use of bone marrow-derived stem cells is effective in avoiding or limiting rejection in allogeneic transplantation (Pino and Humes, 2010). In this study, when compared with the other types of stem cells, ADMSCs (19/72) and MDMSCs (32/72) were easier to obtain, making them most important in research; hence, rich raw data are available for merger analysis. On the contrary, there are few studies on UC-MSCs, FMhMSCs, GPSCs, RPCs, etc. Based on the small amount of data currently available, it is not possible to determine whether the therapeutic effect is different from that of ADMSCs and MDMSCs, also, the true efficacy of other less reported stem cells is not available. In addition, the results of some studies are not fully reported; thus, the sorting results based on the extracted data are only of reference significance, and there may be more potential stem cells.

### Quality of the Evidence

#### Heterogenicity

Good homogeneity in intervention measures, model types, modeling methods and the measurement of outcome indicators of included studies is the premise of using meta-analysis method to synthesize the data in systematic review of animal studies (Ioannidis et al., 2008). There are significant differences in animal species, age, weight, types, sources, and treatment doses of stem cells. In addition, the measurement nodes vary from 18 h (Wang et al., 2013) to 22 wk (Du et al., 2012) after modeling. The criteria for judging the outcome are quite different, including 0–4 (Cai et al., 2014a), 0–5 (Zhou et al., 2017), PAS score system (Tsuda et al., 2014), Millar score system (Huang et al., 2012) and unreported score standard (Havakhah et al., 2018) in terms of renal histopathological score. Therefore, there is a large heterogeneity among the studies, which reduces the credibility of the results of this study to a certain extent, and even draws inconsistent conclusions.

### Internal Authenticity

Among the 72 studies included, the random grouping method of 98.61% (71/72) of the studies was unclear, and none of the studies reported implementation of covert grouping. Additionally, only 44.44% (32/72) of the studies had a balanced baseline characteristic, suggesting the likelihood of selection bias. None of the studies reported blind methods for animal breeders/researchers and outcome evaluators. Although blinding is not necessary in animal studies, and in most studies the researchers are also the animal breeders, it may be necessary to apply blinding to the intervention and the measurement stage of the animal studies to reduce performance and detection bias and increase the authenticity of the results. In addition, qualifications of the surveyors, inconsistencies between different animal models, and the measurement criteria will affect the results to varying degrees (Sessler and Imrey, 2015). However, none of the 72 studies included in this systematic evaluation reported the qualifications of the surveyors or the standards and specific measurement processes used in the study. Although the 72 studies clearly reported all predetermined results in their publications, the original proposals for the studies could not be obtained, and it is impossible to determine whether the results are reported without bias according to the proposals. Selective reporting of experimental animal research results can lead to publication bias, thus affecting the reliability of systematic review conclusions and even prompting an opposite conclusion (Korevaar et al., 2011). In summary, multiple biases such as selective bias, implementation bias measurement bias and reporting bias may exist in the experimental process. These biases are likely to affect the accuracy and internal authenticity of the outcome indicators to a large extent. Furthermore, the evaluation results based on CERqual demonstrated that the quality of evidence of the five outcome indicators was “low” or “very low.” The low quality of the evidence is bound to reduce the reliability of experimental results and the possibility of clinical transformation of the animal experiments.

### External Authenticity

External authenticity refers to the extent to which clinical trial results can be reproduced repeatedly in the target population and in the common population (Wu et al., 2011). The transformation of experimental results from animal studies into clinical trials should focus on the external authenticity regarding the following aspects: *1*) RIRI animal models cannot fully simulate the characteristics of patients with RIRI and therefore cannot completely replace clinical patients; *2*) To avoid many adverse reactions such as immune rejection caused by xenotransplantation, stem cells used in clinical trials are derived from autologous or allogeneic human tissues (Ilic and Polak, 2011)^.^ The types and sources of stem cells included in this study are diverse. Particularly, the transplantation of stem cells from different species may produce immune rejection in animals. *3*) Clinically, a patient’s history and internal or external physical conditions may affect the efficacy of the stem cell therapy, making it difficult to simulate multiple human physical conditions in animal studies; and *4*) The effectiveness of stem cell therapy in animal IRIR models can only be judged by laboratory indicators (e.g., serum creatinine levels), while some outcomes of clinical diseases (e.g., individual subjective experience) cannot be reflected by objective outcome indicators. Due to the above limitations of external authenticity, it is difficult to obtain sufficient evidence to support animal experimental results and conclusions included in this study for the purpose of designing clinical trials.

To sum up, through a comprehensive analysis of the evidence quality from heterogeneity, internal authenticity and external authenticity of the included study, we believe that it is necessary to carefully consider whether the results of each study are reliable reflections of the actual outcomes owing to the limitations of the current animal studies in terms of design methods, results measurement, statistics, and evidence quality. Therefore, based on the analysis of animal studies included in our study, it is still not possible to obtain reliable evidence to determine whether further clinical trials are necessary. However, this study has established that the use of stem cells is a potential treatment modality for RIRI. Among the various stem cells, ADMSCs and MDMSCs appear to have a promising therapeutic potential. However, based on the small number of studies available on other stem cells, their therapeutic potentials cannot be ruled out. Therefore, more high-quality studies are needed to further explore the efficacies of different stem cells in the treatment of RIRI.

### Advantages and Limitations of This Study

This is the first study to systematically evaluate animal studies of stem cell strategies for the treatment of RIRI with certain advantages: *1*) The quality of evidence for each outcome indicator was evaluated using CERQual tools, thus providing a more scientific assessment of the potential for transforming animal experimental studies into clinical trials; *2*) The bias risk of the animal studies was assessed using the internationally recognized SYRCLE’s bias risk assessment tool; and *3*) The internal and external authenticity of the evidence is discussed in detail to objectively assess the feasibility of transforming animal experimental results into clinical practice. The limitations of this systematic review include: *1*) Only Chinese and English databases were queried, which may have led to language bias; and *2*) Grey literature and conference abstracts were not included, which may have led to publication bias.

### Research Outlook

Through the comprehensive analysis of the basic information in each article, the risk of internal bias, the quality of evidence, and the outcomes, the current animal research on stem cell treatment of RIRI may have the following limitations: *1*) Selection of animal models: the current animal model is limited to rats, mice and other rodents, and there are huge differences in anatomical structure, biological characteristics, and mechanisms of disease compared with the human body (Courtine et al., 2007; Bagul et al., 2013). Therefore, it is suggested that we consider the physiological structure of the RIRI disease mechanism in the experimental animals and the differences from RIRI in humans in order to determine the most applicable animal models; *2*) The selection of stem cells: stem cells from the same genus should be selected to avoid adverse reactions such as immunological rejection from xenotransplantation and to improve the stability and reliability of the results; and *3*) The selection of outcome indicators: the safety of drugs or treatments is the first criteria in clinical application followed by effectiveness (Ozdemir et al., 2001). The studies included in this systematic review only measured the efficacy indicators of stem cell therapy; none of the studies reported safety indicators, such as whether stem cells activate granulocytes or aggravate renal injury by affecting the immune system (Tögel et al., 2004). Therefore, future relevant animal studies should also focus on the safety of stem cell therapy to avoid adverse reactions when it is applied to the clinical setting (Bagul et al., 2013; Rota et al., 2019).

In addition to the above issues, preclinical research on stem cell therapy for RIRI should give careful attention to experimental design, implementation, measurement and evaluation of results, and report of the studies, to improve the quality of relevant animal experimental research and to promote the transformation and utilization of its results in human trials.

## Conclusion

Stem cells from different sources can improve renal function in RIRI animals, and ADMSCs and MDMSCs are the most studied and possibly the most potential stem cells for treatment. However, because of the lack of research data on other types of stem cells, it is still not possible to determine the best stem cell therapy. Hence, more experimental studies on the treatment of RIRI with stem cells from multiple sources are warranted. In addition, owing to the bias caused by the limitations of the included studies in the design of experiments, measurement of results, and quality of evidence, the precise efficacies of the stem cells remain unknown. Moreover, there is no reliable evidence to decide on the requirement for further clinical research. Therefore, future animal experiments should be designed in a scientifically sound manner to alleviate the risk of bias and improve the quality of evidence. Such studies are likely to be beneficial in determining the efficacy of stem cells in the treatment of RIRI and in ascertaining the feasibility of clinical transformation so as to reduce the risk of applying pre-clinical results in clinical settings.

## Data Availability

The original contributions presented in the study are included in the article/Supplementary Material, further inquiries can be directed to the corresponding authors.
