# Supplementary material for: Therapeutic Effects of Stem Cells From Different Source on Renal Ischemia- Reperfusion Injury: A Systematic Review and Network Meta-analysis of Animal Studies
Source: Front Pharmacol. 2021 Sep 2;12:713059. doi: 10.3389/fphar.2021.713059 (PMC8444551; doi:10.3389/fphar.2021.713059)
Supplement: Supplementary file 1 [file presentation1.pptx]

## Slide 1
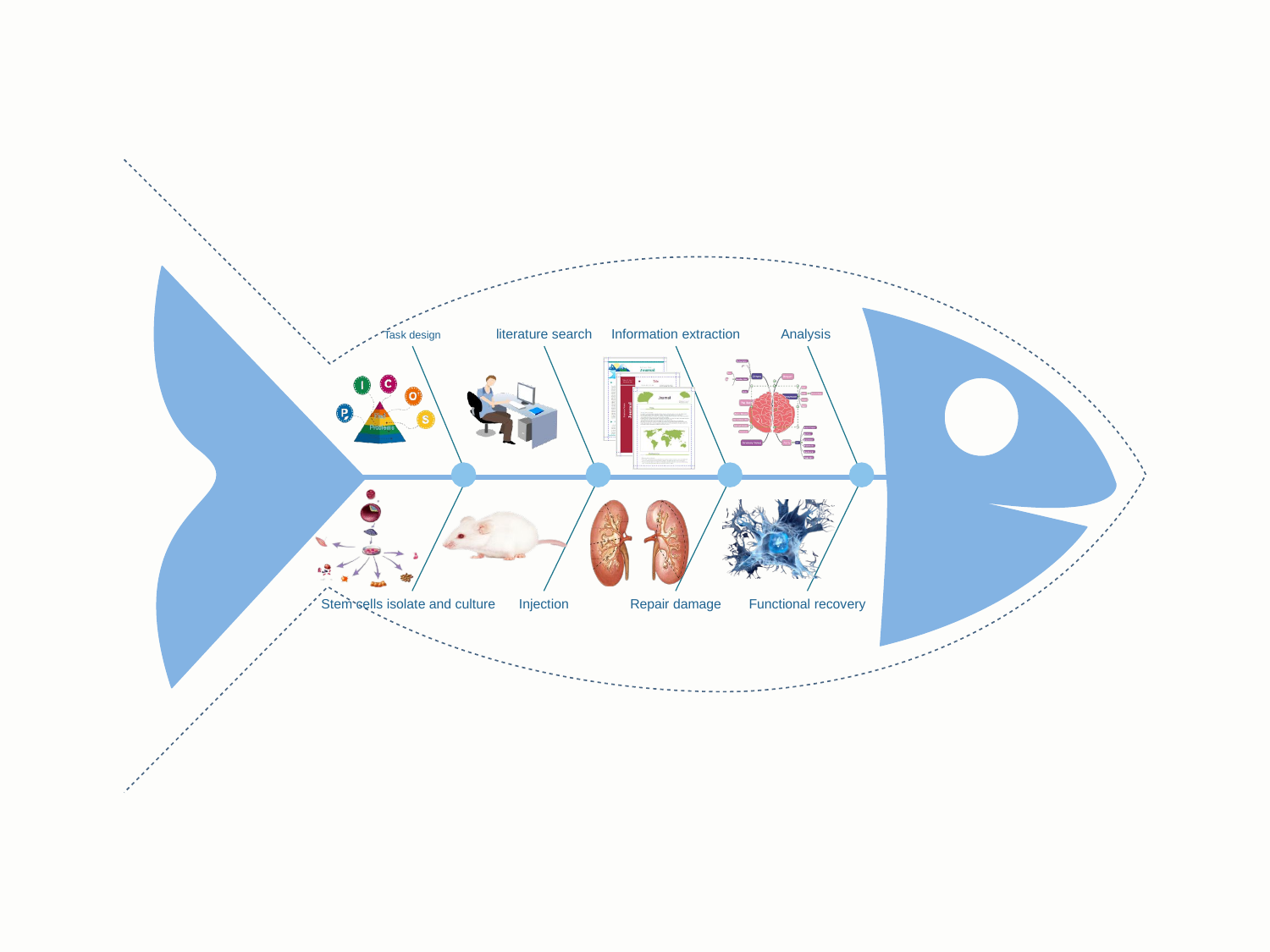

Task design
literature search
Information extraction
Analysis
Stem cells isolate and culture
Injection
Repair damage
Functional recovery
