# Supplementary material for: Therapeutic Effects of Stem Cells From Different Source on Renal Ischemia- Reperfusion Injury: A Systematic Review and Network Meta-analysis of Animal Studies
Source: Front Pharmacol. 2021 Sep 2;12:713059. doi: 10.3389/fphar.2021.713059 (PMC8444551; doi:10.3389/fphar.2021.713059)
Supplement: Supplementary file 2 [file datasheet1.zip › supplementary materials/Annex 1.docx]

**Supplemental data:**

**Chinese and English search strategies**

Comments:

In order for non-Chinese readers to understand the Chinese search strategy of this article, we translated the Chinese search terms in the search formula.

**Pubmed:2021.04**

#1 renal OR kidney OR nephridium(883,188)

#2 "Kidney"[Mesh](354,585)

#3 #1 OR #2(985,397)

#4 ischemia-reperfusion injury OR ischemia reperfusion injury OR ischemia-reperfusion OR reperfusion injury OR ischemia reperfusion(49,137)

#5 "Reperfusion Injury"[Mesh](43,842)

#6 #4 AND #5(62,739)

#7 stem cell OR dry cell OR derived stem cell(173,025)

#8 "Stem Cells"[Mesh](225,219)

#9 #7 AND #8(326,729)

#10 #3 AND #6 AND #9(265)

**Ovid-Embase:1974-2021.04**

#1 (renal or kidney or nephridium).mp. [mp=title, abstract, heading word, drug trade name, original title, device manufacturer, drug manufacturer, device trade name, keyword, floating subheading word, candidate term word](1150434)

#2 exp kidney/(113103)

#3 #1 or #2(1150434)

#4 (ischemia-reperfusion injury or ischemia reperfusion injury or ischemia-reperfusion or reperfusion injury or ischemia reperfusion).mp. [mp=title, abstract, heading word, drug trade name, original title, device manufacturer, drug manufacturer, device trade name, keyword, floating subheading word, candidate term word](78114)

#5 exp reperfusion injury/(51174)

#6 #4 or #5(78114)

#7 (stem cell or dry cell or derived stem cell).mp. [mp=title, abstract, heading word, drug trade name, original title, device manufacturer, drug manufacturer, device trade name, keyword, floating subheading word, candidate term word](533747)

#8 exp stem cell/(128966)

#9 #7 or #8(533747)

#10 #3 and #6 AND #9(611)

**Cochrane library:2021.04**

#1 (renal OR kidney OR nephridium):ti,ab,kw(81411)

#2 MeSH descriptor: [Kidney] explode all trees(3947)

#3 #1 or #2(81421)

#4 (ischemia-reperfusion injury OR ischemia reperfusion injury OR ischemia-reperfusion OR reperfusion injury OR ischemia reperfusion):ti,ab,kw(4167)

#5 MeSH descriptor: [Reperfusion Injury] explode all trees(1117)

#6 #4 or #5(4186)

#7 (stem cell OR dry cell OR derived stem cell):ti,ab,kw(14536)

#8 MeSH descriptor: [Stem Cells] explode all trees(800)

#9 #7 or #8(14626)

#11 #3 and #6 and #9 and #10(10)

**Web of science:2021.04**

TS=(renal OR kidney OR nephridium) AND TS=(ischemia-reperfusion injury OR ischemia reperfusion injury OR ischemia-reperfusion OR reperfusion injury OR ischemia reperfusion) AND TS=(stem cell OR dry cell OR derived stem cell)(901)

**CNKI/高级检索: 2021.04; CNKI / Advanced Search: 2021.04**

主题：(肾 OR 肾脏) AND (缺血再灌注损伤) AND (干细胞 OR 万能细胞) (118)

Subject: (kidney OR renal) AND (ischemia-reperfusion injury) AND (stem cell OR universal cell) (118)

**万方/高级检索: 2021.04; Wanfang database/ Advanced Search: 2021.04**

主题:(肾 OR 肾脏)*主题:(缺血再灌注损伤)*主题:(干细胞 OR 万能细胞) (214)

Subject: (kidney OR renal) AND (ischemia-reperfusion injury) AND (stem cell OR universal cell) (214)

**VIP/高级检索: 2021.04; VIP database/ Advanced Search: 2021.04**

题名或关键词:(肾 OR 肾脏)* 题名或关键词:(缺血再灌注损伤)* 题名或关键词:(干细胞 OR 万能细胞 (5)

Title or keyword: (kidney OR renal) AND (ischemia-reperfusion injury) AND (stem cell OR universal cell) (5)

**CBM/高级检索:2020.01.11; CBM / Advanced Search: 2020.01.11**

#1 ("肾"[常用字段:智能] OR "肾脏"[常用字段:智能]) OR ("肾"[不加权:扩展])(645025)

#2 ("缺血再灌注损伤"[常用字段:智能]) OR ("再灌注损伤"[不加权:扩展])(37243)

#3 ("万能细胞"[常用字段:智能]) OR ("干细胞"[常用字段:智能]) OR ("干细胞"[不加权:扩展])(133728)

#4 ("动物实验"[常用字段:智能] OR "动物"[常用字段:智能] OR "兔"[常用字段:智能] OR "猪"[常用字段:智能] OR "羊"[常用字段:智能] OR "猩猩"[常用字段:智能] OR "蟾蜍"[常用字段:智能] OR "鼠"[常用字段:智能] OR "猴"[常用字段:智能] OR "狗"[常用字段:智能] OR "猿"[常用字段:智能] OR "蛙"[常用字段:智能]) OR ("动物实验"[不加权:扩展])(940298)

#5 #1 AND #2 AND #3 AND #4(97)

#1 (“kidney” [common field: smart] OR “renal” [common field: smart] OR (“renal”[unweighted, extended]) (645025)

#2(“ischemia-reperfusion injury” [common field: smart]) OR (“reperfusion injury” [unweighted, extended]) (37243)

#3(“universal cell” [common field: smart]) OR (“stem cell” [common field: smart]) OR (“stem cell” [unweighted, extended]) (133728)

#4 (“animal experiment” [common field: smart]) OR (“animal” [common field: smart]) OR (“rabbit” [common field: smart]) OR (“pig” [common field: smart]) OR (“sheep” [common field: smart]) OR (“orange” [common field: smart]) OR (“toads” [common field: smart]) OR rat OR (“monkey” [common field: smart]) OR (“dog” [common field: smart]) OR (“apes” [common field: smart]) OR (“frog” [common field: smart]) OR (“animal experiment” [unweighted, extended]) (940298)

#5 #1 AND #2 AND #3 AND #4(97)
