# Supplementary material for: Therapeutic Effects of Stem Cells From Different Source on Renal Ischemia- Reperfusion Injury: A Systematic Review and Network Meta-analysis of Animal Studies
Source: Front Pharmacol. 2021 Sep 2;12:713059. doi: 10.3389/fphar.2021.713059 (PMC8444551; doi:10.3389/fphar.2021.713059)
Supplement: Supplementary file 2 [file datasheet1.zip › supplementary materials/Annex 3.docx]

**Table 1: Traditional Meta-analysis results of serum creatinine levels between stem cell groups and the control group at 1 day after administration**

| Intervention measures | Research quality | SMD [95%CI] | *I^2^*/% | Z | P |
| --- | --- | --- | --- | --- | --- |
| ADMSCs VS Negative control | 12 | -2.164[-3.159, -1.169] | 85.4% | 4.26 | 0.000 |
| MDMSCs VS Negative control | 21 | -1.718[-2.597, -0.838] | 85.8% | 3.38 | 0.000 |
| EPCs VS Negative control | 4 | -5.914[-7.648, -4.180] | 30.1% | 6.69 | 0.232 |
| UC-MSCs VS Negative control | 3 | -5.275[-11.147, 0.597] | 93.7% | 1.76 | 0.000 |
| hAFSCs VS Negative control | 2 | -3.309[-5.350, -1.268] | / | 3.18 | / |
| FMhMSCs VS Negative control | 1 | -3.314[-7.684, 0.879] | 91.0% | 1.49 | 0.001 |
| Fetal Kidney Cells VS Negative control | 1 | 0.166[-0.968, 1.300] | / | 0.29 | / |
| USCs VS Negative control | 1 | -1.877[-3.421, -0.334] | / | 2.38 | / |
| NPCs VS Negative control | 1 | -2.561[-3.773, -1.350] | / | 4.14 | / |
| HAEC VS Negative control | 1 | -2.014[-3.013, -1.015] | / | 3.95 | / |
| SHED VS Negative control | 1 | -0.424[-0.892, 0.043] | / | 1.78 | / |
| RPCs VS Negative control | 1 | -4.174[-5.398, -2.950] | / | 6.68 | / |

**Table 2: Traditional Meta-analysis results of serum creatinine levels between stem cell groups and the control group at 7 days after administration**

| Intervention measures | Research quality | SMD [95%CI] | *I^2^*/% | Z | P |
| --- | --- | --- | --- | --- | --- |
| ADMSCs VS Negative control | 6 | -1.939[-2.541, -1.336] | 31.7% | 6.31 | 0.198 |
| MDMSCs VS Negative control | 7 | -1.407[-2.587, -0.227] | 78.4% | 2.34 | 0.000 |
| EPCs VS Negative control | 2 | -1.969[-3.036, -0.903] | 0.0% | 3.62 | 0.350 |
| USCs VS Negative control | 2 | -1.515[-2.163, -0.867] | 0.0% | 4.58 | 0.842 |
| UC-MSCs VS Negative control | 1 | -0.665[-1.834, 0.503] | / | 1.12 | / |
| SHED VS Negative control | 1 | -0.800[-1.547, -0.052] | / | 2.10 | / |
| RPCs VS Negative control | 1 | -3.130[-4.150, -2.111] | / | 6.02 | / |

**Table 3: Traditional Meta-analysis results of serum creatinine levels between stem cell groups and the control group at 14 days after administration**

| Intervention measures | Research quality | SMD [95%CI] | *I^2^*/% | Z | P |
| --- | --- | --- | --- | --- | --- |
| ADMSCs VS Negative control | 4 | -2.537[-5.120, 0.046] | 87.7% | 1.93 | 0.000 |
| MDMSCs VS Negative control | 4 | -0.780[-2.750, 1.190] | 86.7% | 0.78 | 0.000 |
| EPCs VS Negative control | 2 | -0.774[-1.648, 0.100] | 0.0% | 1.74 | 0.820 |
| USCs VS Negative control | 0 | 0.221[-1.023, 1.465] | / | 0.35 | / |
| RPCs VS Negative control | 0 | -2.000[-2.832, -1.168] | / | 4.71 | / |

**Table 4: Traditional Meta-analysis results of blood urea nitrogen level between stem cell groups and the control group at 1 day after administration**

| Intervention measures | Research quality | SMD [95%CI] | *I^2^*/% | Z | P |
| --- | --- | --- | --- | --- | --- |
| MDMSCs VS Negative control | 16 | -1.715[-2.623, -0.806] | 83.0% | 3.70 | 0.000 |
| ADMSCs VS Negative control | 7 | -2.239[-3.880, -0.597 | 89.0% | 2.67 | 0.000 |
| EPCs VS Negative control | 3 | -2.902[-4.594, -1.211] | 62.7% | 3.36 | 0.068 |
| UC-MSCs VS Negative control | 4 | -3.788[-7.164, -0.412] | 91.1% | 2.20 | 0.000 |
| FMhMSCs VS Negative control | 2 | -2.372[-5.331, 0.587] | 87.5% | 1.57 | 0.005 |
| Fetal Kidney Cells VS Negative control | 1 | -0.474[-1.624, 0.677] | / | 0.81 | / |
| USCs VS Negative control | 1 | -4.185[-6.581, -1.789] | / | 3.42 | / |
| SHED VS Negative control | 1 | -0.479[-0.948, -0.010] | / | 2.00 | / |
| RPCs VS Negative control | 1 | -6.170[-7.824, -4.515] | / | 7.31 | / |

**Table 5: Traditional Meta-analysis results of blood urea nitrogen level between stem cell groups and the control group at 7 days after administration**

| Intervention measures | Research quality | SMD [95%CI] | *I^2^*/% | Z | P |
| --- | --- | --- | --- | --- | --- |
| MDMSCs VS Negative control | 7 | -1.978[-2.513, -1.443] | 0.0% | 7.25 | 0.616 |
| ADMSCs VS Negative control | 2 | -2.210[-3.329, -1.092] | 0.0% | 3.87 | 0.324 |
| EPCs VS Negative control | 2 | -2.076[-4.890, 0.739] | 80.7% | 1.45 | 0.023 |
| USCs VS Negative control | 2 | -0.443[-2.553, 1.666] | 87.8% | 0.41 | 0.004 |
| UC-MSCs VS Negative control | 1 | -0.443[-1.591, 0.705] | / | 0.76 | / |
| SHED VS Negative control | 1 | -0.651[-1.389, 0.086] | / | 1.73 | / |
| RPCs VS Negative control | 1 | -0.102[-0.775, 0.570] | / | 0.3 | / |

**Table 6: Traditional Meta-analysis results of blood urea nitrogen level between stem cell groups and the control group at 14 days after administration**

| Intervention measures | Research quality | SMD [95%CI] | *I^2^*/% | Z | P |
| --- | --- | --- | --- | --- | --- |
| ADMSCs VS Negative control | 3 | 3.483[-2.649, 9.616] | 94.6% | 1.11 | 0.000 |
| MDMSCs VS Negative control | 4 | 2.377[-2.686, 7.441] | 92.2% | 0.92 | 0.000 |
| EPCs VS Negative control | 2 | 10.857[5.227, 16.488] | 49.7% | 3.78 | 0.159 |
| USCs VS Negative control | 1 | 18.871[9.541, 28.200] | / | 3.96 | / |
| RPCs VS Negative control | 1 | -1.072[-1.793, -0.350] | / | 2.91 | / |

**Table 7: Traditional Meta-analysis results of histological scores between stem cell groups and the control group in early stage after administration**

| Intervention measures | Research quality | SMD [95%CI] | *I^2^*/% | Z | P |
| --- | --- | --- | --- | --- | --- |
| MDMSCs VS Negative control | 5 | -1.598[-2.728, -0.469] | 72.5% | 3.52 | 0.006 |
| ADMSCs VS Negative control | 2 | -4.116[-8.005, -0.228] | 86.4% | 2.77 | 0.007 |
| EPCs VS Negative control | 1 | -3.289[-5.121, -1.456] | / | 2.07 | / |
| FMhMSCs VS Negative control | 1 | -10.097[-15.975, -4.218] | / | 3.37 | / |
| SHED VS Negative control | 1 | -0.615[-1.088, -0.142] | / | 2.55 | / |

**Table 8: Traditional Meta-analysis results of proliferation of resident cells between stem cell groups and the control group in early stage after administration**

| Intervention measures | Research quality | SMD [95%CI] | *I^2^*/% | Z | P |
| --- | --- | --- | --- | --- | --- |
| ADMSCs VS Negative control | 6 | 4.282 [-2.734, 5.829] | 69.0% | 5.42 | 0.006 |
| MDMSCs VS Negative control | 5 | 3.138[0.530, 5.746] | 85.0% | 2.36 | 0.000 |
| USCs VS Negative control | 1 | 0.240[-1.005, 1.485] | / | 0.38 | / |
| iPSCs VS Negative control | 1 | 5.425[3.330, 7.519] | / | 5.08 | / |
| RPCs VS Negative control | 1 | 2.156[1.301, 3.012] | / | 4.94 | / |

**Table 9: Traditional Meta-analysis results of** **apoptosis of resident cells between stem cell groups and the control group in early stage after administration**

| Intervention measures | Research quality | SMD [95%CI] | *I^2^*/% | Z | P |
| --- | --- | --- | --- | --- | --- |
| ADMSCs VS Negative control | 3 | -5.661[-7.104, -4.219] | 0.0% | 7.69 | 0.923 |
| MDMSCs VS Negative control | 2 | -0.569[-1.578, 0.441] | 0.0% | 1.10 | 0.743 |
| USCs VS Negative control | 2 | -4.408[-12.502, 3.685] | 91.7% | 1.07 | 0.001 |
| FMhMSCs VS Negative control | 1 | -23.527[-36.911, 10.144] | / | 3.45 | / |
| Fetal Kidney Cells VS Negative control | 1 | -3.863[-5.900, -1.827] | / | 3.72 | / |
| iPSCs VS Negative control | 1 | -6.019[-8.299, -3.738] | / | 5.17 | / |
| HAEC VS Negative control | 1 | -7.904[-10.374, -5.434] | / | 6.27 | / |
